# Functional DNA methylation signatures for autism spectrum disorder genomic risk loci: 16p11.2 deletions and *CHD8* variants

**DOI:** 10.1186/s13148-019-0684-3

**Published:** 2019-07-16

**Authors:** M. T. Siu, D. T. Butcher, A. L. Turinsky, C. Cytrynbaum, D. J. Stavropoulos, S. Walker, O. Caluseriu, M. Carter, Y. Lou, R. Nicolson, S. Georgiades, P. Szatmari, E. Anagnostou, S. W. Scherer, S. Choufani, M. Brudno, R. Weksberg

**Affiliations:** 1 Program in Genetics and Genome Biology, The Hospital for Sick Children, Toronto, Ontario Canada; 2 Centre for Computational Medicine, The Hospital for Sick Children, Toronto, Ontario Canada; 3 Division of Clinical and Metabolic Genetics, The Hospital for Sick Children, 555 University Ave, Toronto, Ontario M5G 1X8 Canada; 4 Department of Molecular Genetics, University of Toronto, Toronto, Ontario Canada; 5 Pediatric Laboratory Medicine, The Hospital for Sick Children, Toronto, Ontario Canada; 6 Laboratory Medicine and Pathobiology, University of Toronto, Toronto, Ontario Canada; 7 The Centre for Applied Genomics, The Hospital for Sick Children, Toronto, Ontario Canada; 8 Department of Medical Genetics, University of Alberta, Edmonton, Alberta Canada; 9 Department of Genetics, The Children’s Hospital of Eastern Ontario, Ottawa, Ontario Canada; 10 Department of Psychiatry, University of Western Ontario, London, Ontario Canada; 11 Department of Psychiatry and Behavioural Neurosciences, Offord Centre for Child Studies, McMaster University, Hamilton, Ontario Canada; 12 Child and Youth Mental Health Collaborative, Centre for Addiction and Mental Health, The Hospital for Sick Children, Toronto, Ontario Canada; 13 Department of Psychiatry, University of Toronto, Toronto, Ontario Canada; 14 Holland Bloorview Kids Rehabilitation Hospital, Toronto, Ontario Canada; 15 Department of Pediatrics, University of Toronto, Toronto, Ontario Canada; 16 Department of Computer Science, University of Toronto, Toronto, Ontario Canada; 17 Institute of Medical Science, School of Graduate Studies, University of Toronto, Toronto, Ontario Canada

**Keywords:** Epigenetics, DNA methylation, Autism spectrum disorder, Genomic variants, Genetic stratification, Heterogeneity

## Abstract

**Background:**

Autism spectrum disorder (ASD) is a common and etiologically heterogeneous neurodevelopmental disorder. Although many genetic causes have been identified (> 200 ASD-risk genes), no single gene variant accounts for > 1% of all ASD cases. A role for epigenetic mechanisms in ASD etiology is supported by the fact that many ASD-risk genes function as epigenetic regulators and evidence that epigenetic dysregulation can interrupt normal brain development. Gene-specific DNAm profiles have been shown to assist in the interpretation of variants of unknown significance. Therefore, we investigated the epigenome in patients with ASD or two of the most common genomic variants conferring increased risk for ASD. Genome-wide DNA methylation (DNAm) was assessed using the Illumina Infinium HumanMethylation450 and MethylationEPIC arrays in blood from individuals with ASD of heterogeneous, undefined etiology (*n* = 52), and individuals with 16p11.2 deletions (16p11.2del, n = 9) or pathogenic variants in the chromatin modifier *CHD8* (*CHD8*^+/−^, n = 7).

**Results:**

DNAm patterns did not clearly distinguish heterogeneous ASD cases from controls. However, the homogeneous genetically-defined 16p11.2del and *CHD8*^*+/−*^ subgroups each exhibited unique DNAm signatures that distinguished 16p11.2del or *CHD8*^*+/−*^ individuals from each other and from heterogeneous ASD and control groups with high sensitivity and specificity. These signatures also classified additional 16p11.2del (*n* = 9) and *CHD8* (*n* = 13) variants as pathogenic or benign. Our findings that DNAm alterations in each signature target unique genes in relevant biological pathways including neural development support their functional relevance. Furthermore, genes identified in our *CHD8*^*+/−*^ DNAm signature in blood overlapped differentially expressed genes in *CHD8*^*+/−*^ human-induced pluripotent cell-derived neurons and cerebral organoids from independent studies.

**Conclusions:**

DNAm signatures can provide clinical utility complementary to next-generation sequencing in the interpretation of variants of unknown significance. Our study constitutes a novel approach for ASD risk-associated molecular classification that elucidates the vital cross-talk between genetics and epigenetics in the etiology of ASD.

**Electronic supplementary material:**

The online version of this article (10.1186/s13148-019-0684-3) contains supplementary material, which is available to authorized users.

## Introduction

Autism spectrum disorder (ASD) comprises a group of complex neurodevelopmental conditions, diagnosed in 1 of 68 (1.4%) individuals in the general population [1]. Significant etiologic heterogeneity is implied by the variable penetrance of a large number of genetic causes involving > 200 ASD-risk loci (i.e., single genes, copy number variants [CNVs]) that have been identified in ~ 25–40% of individuals with ASD [2–6]; no single genetic cause accounts for > 1% of ASD cases [6–9]. Given that ASD is a multifactorial disorder, the expectation is that the combined contribution of multiple etiologic factors will determine an individual’s risk, whereby once a threshold is reached, the clinical features expressed will support a diagnosis of ASD [10]. Other etiologic factors currently implicated in ASD etiology include environmental and epigenetic mechanisms. Many ASD-risk genes are “epigenes” encoding proteins that function as epigenetic regulators, i.e., chromatin remodelers and transcriptional regulators, supporting a role for epigenetic dysregulation in ASD etiology [4, 11–13]. Epigenetic marks such as DNA methylation (DNAm) are precisely programmed spatially and temporally during normal development. DNAm alterations caused by genetic and/or environmental factors could negatively impact biological pathways important for normal brain development.

DNAm is one of the most stable and commonly assessed epigenetic marks. DNAm results from the transfer of a methyl group to cytosine in a CpG dinucleotide, catalyzed by DNA methyltransferase enzymes. These modifications are established and maintained through cell division. We, and others, have shown that there is crosstalk between histone modifications and CpG methylation in DNA; that is, cells from individuals with variants in some epigenes demonstrate functionally relevant genome-wide DNAm alterations that constitute gene-specific signatures. DNAm signatures have been previously identified in peripheral blood for disorders caused by pathogenic variants in epigenes that are associated with neurodevelopmental outcomes including both non-syndromic ASD and syndromic ASD/intellectual disability (ID) (e.g., *NSD1* variants associated with Sotos syndrome, *CHD7* variants associated with CHARGE syndrome) [14–23]. We have shown that these DNAm signatures represent a promising avenue to improve an increasingly challenging issue in clinical diagnostics. While advancing clinical diagnostics, next-generation sequencing (NGS) has magnified one of the most significant challenges of DNA sequencing-based testing, namely the interpretation of variants of unknown significance (VUS). VUS are increasingly detected as the utilization of diagnostic sequencing expands in both the clinical and research arenas, also true in the context of ASD. Specifically, DNAm signatures are proving to be effective in the functional classification of VUS [14, 15, 18, 19]. A limited number of studies have examined the potential for developing ASD-specific DNAm biomarkers [24–29]. They report inconsistent differences at a variety of genomic sites in peripheral blood [29], brain [26, 27, 30], buccal epithelium [24], and sperm [31]. These studies are limited by small sample sizes and modest DNAm differences between ASD cases and controls likely due not only to inter-tissue and cell type variability, but also more importantly to heterogeneity of DNAm alterations in individuals with ASD with unselected etiologies.

We hypothesized that epigenetic alterations contribute to the molecular etiology of ASD and that the discovery of such epigenetic alterations could be enhanced by genetic stratification of ASD cases. More specifically, we expected that individuals with ASD and variants in specific epigenes would display distinct DNAm signatures, as previously demonstrated for other human disorders caused by variants in epigenes or CNVs overlapping epigenes [14–17, 20, 21, 23]. We also expect that we will be able to use the DNAm signatures to classify additional cases with variants in the specific epigenes or CNVs overlapping epigenes as pathogenic or benign. We therefore undertook several analyses comparing ASD cases and controls. First, we compared genome-wide DNAm in blood from individuals with ASD of undefined etiology (i.e., heterogeneous ASD) to controls. Next, we compared controls to cases with two of the most common genomic variants associated with an increased risk for ASD.

The first genetically homogeneous subgroup comprised individuals with 600 kb deletions in 16p11.2, a CNV known to confer ASD susceptibility (16p11.2del; OMIM# 611913). Individuals with 16p11.2del have a variable phenotype; 20–30% receive a diagnosis of ASD, although the majority of these individuals have behavioral/neurocognitive ASD features [32–34]. The HIRA*-*interacting protein 3 gene (*HIRIP3)*, located in the 16p11.2del region, is known to be involved in regulating histone metabolism and chromatin function [35], but the full impact of 16p11.2 deletions on epigenetic dysregulation has yet to be elucidated. The second genetically homogeneous subgroup comprised individuals with known pathogenic variants in the chromodomain helicase DNA-binding protein 8 gene *(CHD8*^*+/−*^; OMIM# 615032). More than 85% of individuals with *CHD8* variants have ASD, as well as morphological anomalies and other neurodevelopmental features [4, 36, 37]. *CHD8* is known to function as an epigenetic regulator, playing a critical role in transcriptional repression through chromatin remodeling [38].

Our results demonstrate that genetically defined subgroups of cases with genomic aberrations at ASD-risk loci involving epigenes have highly sensitive and specific DNAm signatures that distinguish them from heterogeneous ASD cases and controls. We also show that these DNAm signatures identify genomic targets that may contribute to the etiology of specific genetic subgroups of ASD. The differentially methylated gene targets of our DNAm signatures function in biological pathways that are relevant to proposed pathophysiological mechanisms underlying ASD, highlighting the biological significance of our DNAm signatures. Lastly, we show that our DNAm signatures have the potential to be used as a complementary molecular diagnostic tool to help interpret the pathogenicity of VUS, demonstrating their functional utility and potential for clinical application.

## Methods and materials

### Participants

The ASD group consisted of an etiologically undefined, heterogeneous sample of individuals (heterogeneous ASD; *n* = 52) not selected for any known genomic features, for whom deep clinical phenotyping and whole genome/exome sequencing data were available through the Province of Ontario Neurodevelopmental Disorders (POND) Network and the Simons Simplex Collection (SSC) (Additional file 3: Table S1). All neurotypical control samples were selected from a collection available in our laboratory (Additional file 3: Table S1). The majority of individuals included in this study are of Caucasian descent; however, ethnicity data were not available for all individuals included. Some individuals in the control group received formal cognitive/behavioral assessments (Dr. Greg Hanna, University of Michigan); others were recruited using physician/parental screening questionnaires. Our 16p11.2del training case group consisted of DNA samples from the blood of individuals with confirmed typical 600 kb deletions in 16p11.2 (16p11.2del; chr16: 29.5–30.1 Mb) that were used for signature derivation (*n* = 9; 3 with ASD diagnosis) (Table 1). The 16p11.2 test case group consisted of samples from additional individuals with 16p11.2del variants and were used to test the signature (classification) (*n* = 9; 4 with ASD diagnosis) (Hospital for Sick Children [SickKids], POND; Table 3). Our *CHD8*^*+/−*^ training case group consisted of DNA samples from the blood of individuals with confirmed de novo *CHD8* pathogenic variants (*CHD8*^*+/−*^; *n* = 7, all with ASD diagnosis) obtained from SSC (Table 2) for signature derivation. The *CHD8*^*+/−*^ test case group consisted of additional *CHD8* sequence variants (*n* = 13; 12 with an ASD diagnosis) for classification. These were obtained from SSC, SickKids, The University of Alberta (Dr. Oana Caluseriu), and the Children’s Hospital of Eastern Ontario (CHEO; Dr. Melissa Carter) (Table 3).

**Table 1:** Demographic and deletion information for 16p11.2del training signature cases and age-, sex-matched controls

Sample ID	Sex	Age (years)	ASD status	16p11.2del (600 kb region) coordinates	Deletion size (kb)	Inheritance
435	M	12	Unknown	29,656,657–30,190,593	534	Unknown
516	M	7	Unknown	29,656,657–30,190,593	534	Unknown
639	F	13	Unknown	29,592,751–30,190,593	598	Unknown
797	M	7	Unknown	29,590,493–30,190,593	600	Unknown
871	M	10	Unknown	29,590,493–30,190,593	600	Unknown
293	M	5	Unknown	29,567,295–30,178,406	611	Unknown
3-0269-000	M	4	ASD	29,567,309–30,177,807	610	Maternal
1-0019-004	M	7.5	ASD	29,652,799–30,199,507	547	De novo
7-0229-003	M	11.5	ASD	29,567,295–30,177,916	611	Unknown
EP15W	M	13	Control			
EP16W	F	2	Control			
EP17W	F	4	Control			
EP1W	F	8	Control			
EP22W	M	4	Control			
EP2W	F	10	Control			
EP3W	F	13	Control			
EP4W	M	13	Control			
EP5W	F	8	Control			
EP6W	M	13	Control			
EP7W	M	9	Control			
EP8W	M	8	Control			
EP9W	M	11	Control			
EP51	M	12	Control			
EP60W	M	12	Control			
460/2048	M	11.5	Control			
EP32W	M	6	Control			
EP45W	M	10	Control			
EP59W	M	6	Control			
EP57W	M	7	Control			
CT0011-003	M	4	Control			
MK-311/1601	M	10	Control			
KA-352/1734	M	7	Control			

Mean age 16p11.2del 8.556 ± 3.196

Mean age Control 8.761 ± 3.35

16p11.2.del M, F 8, 1

Control M, F 17, 6

**Table 2:** Demographic and variant information for *CHD8*^*+/−*^ training signature cases and age-, sex-matched controls

Sample ID	Sex	Age (years)	ASD status	Nucleotide change	Amino acid change	Exon	Inheritance
11654.p1	F	12.5	ASD	c.3517 A>G	p.Arg1173Gly	16	De novo
12714.p1	M	5	ASD	c.185C>G	p.Ser62X	1	De novo
12752.p1	F	8	ASD	c.6359_6360del	p.Leu2120ProfsX	32	De novo
12991.p1	M	16	ASD	c.6307_6308del	p.Glu2103ArgfsX	31	De novo
13844.p1	M	5	ASD	c.3712C>T	p.Gln1238X	17	De novo
14016.p1	M	14	ASD	c.4009C>T	p.Arg1337X	19	De novo
14233.p1	M	10	ASD	c.7113_7114del	p.Asn2371LysfsX2	36	De novo
EP48W	F	11	Control				
EP58W	M	15	Control				
EP41W	M	16	Control				
EH-268 W	F	6	Control				
JS-265 W	M	16	Control				
EP34W	F	13	Control				
TG-278 W	M	13	Control				
TB-342/1706	M	16	Control				
EP43W	F	7	Control				
EP32W	M	6	Control				
JL-137/1362	M	14	Control				
EP45W	M	10	Control				
EP59W	M	6	Control				
EP57W	M	7	Control				
CT0009-003	M	8	Control				
CT0011-003	M	4	Control				
MK-311/1601	M	10	Control				
EP39W	F	13	Control				
EP42W	F	8	Control				
KA-352/1734	M	7	Control				
MM-119/1567	M	10	Control				

Mean age *CHD8*^*+/−*^ 10.07 ± 4.325

Mean age Control 10.29 ± 3.836

*CHD8*^*+/−*^ M, F 5, 2

Control M, F 15, 6

**Table 3:** Variant details and respective classification scores for 16p11.2del CNV and *CHD8* test cases

Sample ID	Sex	Age (years)	16p11.2del coordinates (size in kb)	Diagnostic classification for 16p11.2 typical deletion (pathogenic, benign, variant of unknown significance [VUS])	Inheritance	ASD status	16p11.2del score
348	M	0.25	29,590,493–30,190,593 (600)	Pathogenic	Unknown	Unknown	0.11505
228	M	0.4	29,590,493–30,190,593 (600)	Pathogenic	Unknown	Unknown	0.12668
875	F	4	29,656,657–30,190,593 (534)	Pathogenic	Unknown	Unknown	0.03073
172	F	6	29,590,493–30,190,593 (600)	Pathogenic	Unknown	Unknown	0.04668
292	M	0.25	29,500,252–30,098,094 (598)	Pathogenic	Unknown	Unknown	0.13978
2-0088-003	F	12	29,652,799–30,199,507 (547)	Pathogenic *(MOSAIC)*	De novo	ASD	-0.00441
3-0406-000	M	1.2	29,581,764–30,199,579 (618)	Pathogenic	Unknown	ASD	0.07735
6-0196-03	M	Unknown	29,500,084–30,098,210 (598)	Pathogenic	Unknown	ASD	0.04815
1-0616-003	M	7.5	28,751,255–28,952,351 (201)	Not determined*	Maternal	ASD	− 0.11936
Sample ID	Sex	Age (years)	*CHD8* variant nucleotide change; amino acid change (Exon #)	Diagnostic classification (pathogenic, benign, variant of unknown significance [VUS])	Inheritance	ASD status	*CHD8*^*+/−*^ score
14406.p1	M	13	c.7493_7495del; p.His2498del (37)	VUS	De novo	ASD	− 0.03482
1-0494-003	M	4.75	c.2219A>G; p.Asn740Ser (9)	Benign	Paternal	ASD	− 0.04451
2-1228-003	F	4.5	c.6841C>G; p.Pro2281Ala (34)	Benign	Paternal	ASD	− 0.04178
1-0277-003	M	Unknown	c.6565G>A; p.Gly2189Arg (33)	Benign	Maternal	ASD	− 0.03065
1-0507-003	F	14	c.3940G>A; p.Ala1314Thr (19)	Benign	Maternal	ASD	− 0.05507
7-0167-003	M	4	c.7499A>C; p.His2500Pro (37)	Benign	Unknown	ASD	− 0.05984
1-0559-003	M	7.7	c.6148dupA; p.Thr2050fs (31)	Pathogenic	De novo	ASD	0.0437
*CHD8*-V1	M	10	c.4215G>T; Synonymous (21)	VUS	Paternal	ASD	− 0.04478
*CHD8*-V2 (father of *CHD8-V1*)	M	40	c.4215G>T; Synonymous (21)	VUS	Not applicable	Unaffected	− 0.04332
CHD8-V3**	M	6.5	c.6649C>T, p.Arg2217* (33)	Pathogenic	Paternal	ASD	0.04616
CHD8-V4 (sibling of CHD8-V3)**	M	14	c.6649C>T, p.Arg2217* (33)	Pathogenic	Paternal	ASD	0.03631
DW0013**	M	13.5	c.6947delC, p.Pro2316Leufs*39 (35)	Likely pathogenic	Unknown	ASD	0.05183
EX0070-W**	M	6.75	c.4327C>T, p.Arg1443Cys (21)	VUS	Paternal	ASD	− 0.04131

*Deletion encompassing *SH2B1* gene, not overlapping 16p11.2del typical deletion region

**Test case data are from EPIC array, classified using 93 probes overlapping 450K and EPIC arrays from *CHD8*^*+/−*^ signature

### DNAm analysis

DNA was extracted from whole blood for all cases and controls using standard techniques. Extracted DNA was sodium bisulfite converted using the Qiagen EZ DNA Methylation kit (Qiagen, Valencia, CA), according to the manufacturer’s protocol. All DNA samples were processed according to the manufacturer’s protocol for DNAm analysis using either the Illumina Infinium HumanMethylation450 BeadChip array (450K array) or the Illumina MethylationEPIC (EPIC array; an additional set of *CHD8* test case sequence variants only) at The Centre for Applied Genomics (SickKids). The distribution of the samples on the arrays was randomized for all cases and controls and for age and sex variables. The 450K array covers > 485,000 individual CpG sites, whereas the EPIC array covers > 850,000 individual CpG sites genome-wide at single-nucleotide resolution, encompassing > 99% of RefSeq genes and > 96% of annotated CpG islands.

### Statistical analysis: DNAm signatures for 16p11.2del and *CHD8*^*+/−*^

Raw genome-wide DNAm data were analyzed using our laboratory’s bioinformatics pipeline employing hierarchical clustering, principal component analysis (PCA) and non-parametric statistical comparisons [15] (Additional file 2: Figure S1). Raw data normalization and pre-processing quality control steps were performed to ensure that only high-quality data were included for group comparisons to identify DNAm differences. Illumina Genome Studio software was used to normalize data (IDAT files) using background subtraction and control normalization to generate methylation values (Beta [*β*]-values). Before analysis, data for probes located on sex chromosomes, autosomal probes that cross-react with sex chromosome probes, non-specific probes, and probes targeting CpG sites within 5 bp of a SNP with a minor allele frequency > 1% were removed [16] (Additional file 3: Table S2). For signature derivation, batch correction by mean-centering was applied in Qlucore Omics Explorer (QOE, www.qlucore.com) to the comparison of 16p11.2del cases to age- (Mann-Whitney *U* test, *p* > 0.05), sex-matched (Chi-square (*χ*^2^) test, *p* > 0.05) controls before performing group comparisons; all other case-control groups were run in single batches. Since there were differences in the composition of age and sex in each group (ASD, 16p11.2del, and *CHD8*^*+/−*^), each training group was compared to a different set of age- and sex-matched controls. The topmost variable probes were used for group comparisons (i.e., least variable CpG sites across the dataset were removed using the QOE 3.2 “filtering by variance” tool), a commonly used prioritization step that increases statistical power while being blinded to group assignment [39]. For example, a variance-filtering parameter “var = 0.02” removes sites with a standard deviation < 2% of the maximal standard deviation. The number of sites retained depended on the specific samples included in each paired group comparison. The derivation of DNAm signatures employed the use of *limma* regression modeling to account for covariates and the Mann-Whitney *U* test to account for possible non-parametric effects. Significantly differentially methylated sites between signature training cases and controls were first identified using *limma* regression, using age, sex, and estimated cell type proportion (*minfi*) as covariates, with a Benjamini-Hochberg adjusted *p* value (*q*-value) < 0.05 and requiring a |Δβ| ≥ 0.05 (at least 5% methylation difference). We then identified significant differentially methylated sites independently using the non-parametric Mann-Whitney *U* test with a Benjamini-Hochberg adjusted *p* value (*q*-value) < 0.05. Optimal *q*-value and DNAm effect size (Δβ) thresholds were selected using volcano plots (Additional file 2: Figures S2 and S3). To be conservative, the final DNAm signature CpG lists were defined as the overlap among the results of *limma* regression, the Mann-Whitney *U* test, and the effect-size requirement.

In addition to incorporating blood cell type proportions as covariates in the derivation of our DNAm signatures (Additional file 1) [40], DNAm data for different blood cell-subtypes [41] were also used to directly ensure that differential methylation at signature sites was not confounded by differences in blood cell-type proportions, as similarly applied in Ref. [14, 15] (Supplementary Information; Additional file 2: Figures S5 and S6).

### Comparison of differentially methylated CpG sites across DNAm signatures and differentially methylated regions (DMRs)

To detect consistent patterns of DNAm alterations from our DNAm signatures, we used an additional method to identify significantly differentially methylated regions (DMRs) overlapping and extending from individual CpG sites contained in each respective DNAm signature. We used the bump hunting method [42], which strengthens the detection of regional differences by combining differential methylation patterns across neighboring CpG sites [43]. The analysis detected DMRs that comprise CpGs with |Δβ| ≥ 5% between cases and controls as candidates for the DMRs, with gaps ≤ 500 bp between neighboring CpGs. Statistical significance was established using 1000 randomized bootstrap iterations to account for confounders, as recommended in the Bioconductor *bumphunter* package documentation. The resulting DMRs were post-filtered to retain only those with *p* values < 0.05 and average methylation differences |Δβ| ≥ 5% across the DMR. To further enhance robustness and improve stringency, we also required these DMRs to (1) comprise at least three consecutive CpGs and (2) include at least one CpG from the DNAm signatures previously derived for either 16p11.2del or *CHD8*^*+/−*^, as described above (Additional file 3: Tables S3 and S4). DMRs are presented using visualization methods adapted from DMRcate software (Additional file 2: Figure S4) [44].

### Testing sensitivity and specificity of 16p11.2del and *CHD8*^*+/−*^ DNAm signatures using independent test cases and controls

The 16p11.2del and *CHD8*^*+/−*^-specific DNAm signatures were used to build predictive models to functionally classify independent 16p11.2del CNVs and *CHD8* test case variants as well as unrelated controls as pathogenic or benign. These include variants that were reported as pathogenic, benign, or of unknown significance (VUS). To evaluate the predictive models, we used independent, unrelated test cases to determine sensitivity and controls to determine specificity. We evaluated sensitivity on a test cohort of 16p11.2del CNV (*n* = 9) and *CHD8* variants (*n* = 13), independent of the training cases. Four of the *CHD8* variant test cases were run on the EPIC array. These test cases were classified using the 450K-derived *CHD8*^*+/−*^ DNAm signature probes that overlapped with those on the EPIC array (92/103 signature sites). To evaluate the specificity of the DNAm classification signatures, we tested the DNAm signatures on a test set of control 450K array data (*n* = 162, described in further detail below), independent of those used in the signature derivation. To confirm that the two models did not generate overlapping predictions, we applied the 16p11.2del predictive model to the *CHD8*^*+/−*^ cohort, and conversely the *CHD8*^*+/−*^ predictive model to the 16p11.2del cohort. For each set of training cases and controls used to derive a signature, a reference DNAm profile was generated across the signature-specific CpGs, by computing median DNAm for each CpG site over all samples in the selected cohort (separately for cases and controls) [15]. Classification scores for additional variant test cases and control samples (Table 2; Additional file 3: Table S5) and corresponding classification plots were generated by comparing the correlation of each sample to the two respective reference profiles (corresponding to signature training cases and to controls). For example, blood samples with positive 16p11.2del scores will cluster more closely with the 16p11.2del reference profile, whereas samples with negative 16p11.2del scores will cluster more closely with the control reference profile.

### Genomic distribution of DNAm signature CpG sites

The genomic distribution of CpG sites contained in each DNAm signature was compared with that of the respective variance-filtered datasets to identify signature-specific regions of enrichment or depletion using a hypergeometric test (implemented in R).

### Additional datasets used for investigating functional significance of DNAm signatures

For additional specificity analysis, we used an independent publicly available set of 450K DNAm data derived from whole blood of control individuals (*n* = 162) with known ages < 50 years (mean 35.8 years) (Gene Expression Omnibus (GEO) Database; www.ncbi.nlm.nih.gov/geo/) (Additional file 3: Table S5). We did not expect control individuals to have 16p11.2del or *CHD8* variants as these genomic variants are present in < 0.04% of the general population [45–47].

To determine the biological relevance of our DNAm signatures, we compared our differentially methylated genes with known ASD-risk genes (SFARI Gene [https://gene.sfari.org/]). To examine cross-tissue and functional relevance of our *CHD8*^*+/−*^ signature, we compared the list of differentially methylated genes to those differentially expressed in an independent study using an engineered hemizygous deletion of *CHD8* (*CHD8*^*+/−*^*;* using CRISPR/Cas9) in human induced pluripotent stem cell (iPSC)-derived neuronal precursor cells (NPCs), differentiated neurons [48], and cerebral organoids [49].

We used GREAT 3.0.0 (http://great.stanford.edu; up to 1 Mbp extension from the nearest genes) to identify functional enrichment of biological pathways in the differentially methylated genes of each DNAm signature in the context of wider genomic regions. Enrichment of each set of signature CpGs was defined against the background set of all probes that remained in the data after filtering by variance.

### DNAm validation by targeted sodium bisulfite pyrosequencing

Specific differentially methylated sites found in each respective DNAm signature for 16p11.2del or *CHD8*^*+/−*^ training signature cases and their age-, sex-matched controls were validated on sodium bisulfite-converted DNA using targeted pyrosequencing assays. These assays were designed using Qiagen Assay Design Software v2.0 to target specific CpGs identified by the microarray experiment as well as adjacent sites (Additional file 1). Selected sites are significantly differentially methylated in both the respective DNAm signatures and DMRs (Additional file 2: Figure S4; Additional file 3: Tables S3 and S4). Pyrosequencing was performed using the PyroMark Q24 system and Pyrosequencing Gold Reagents (Qiagen). Testing for a statistical difference between groups was performed using a Mann-Whitney *U* test (*p* < 0.05).

## Results

### DNAm does not distinguish heterogeneous ASD cases from controls

DNAm profiles of the heterogeneous ASD group (*n* = 52) were compared with age-, sex-matched controls (*n* = 30) at 40550 sites following filtering by variance. Neither *limma* nor Mann-Whitney *U* analyses identified any significant sites after Benjamini-Hochberg correction for multiple testing (all *q* > 0.05). Only 22 sites (16 (72.3%) hypomethylated, 6 (27.3%) hypermethylated) passed our criteria of an unadjusted *p* < 0.001 and |Δβ| > 5%. At this set of sites, no clear distinction between the ASD cases and controls could be made by either hierarchical clustering or principal components analysis (PCA), and thus no DNAm signature was identified.

**Fig. 1:**
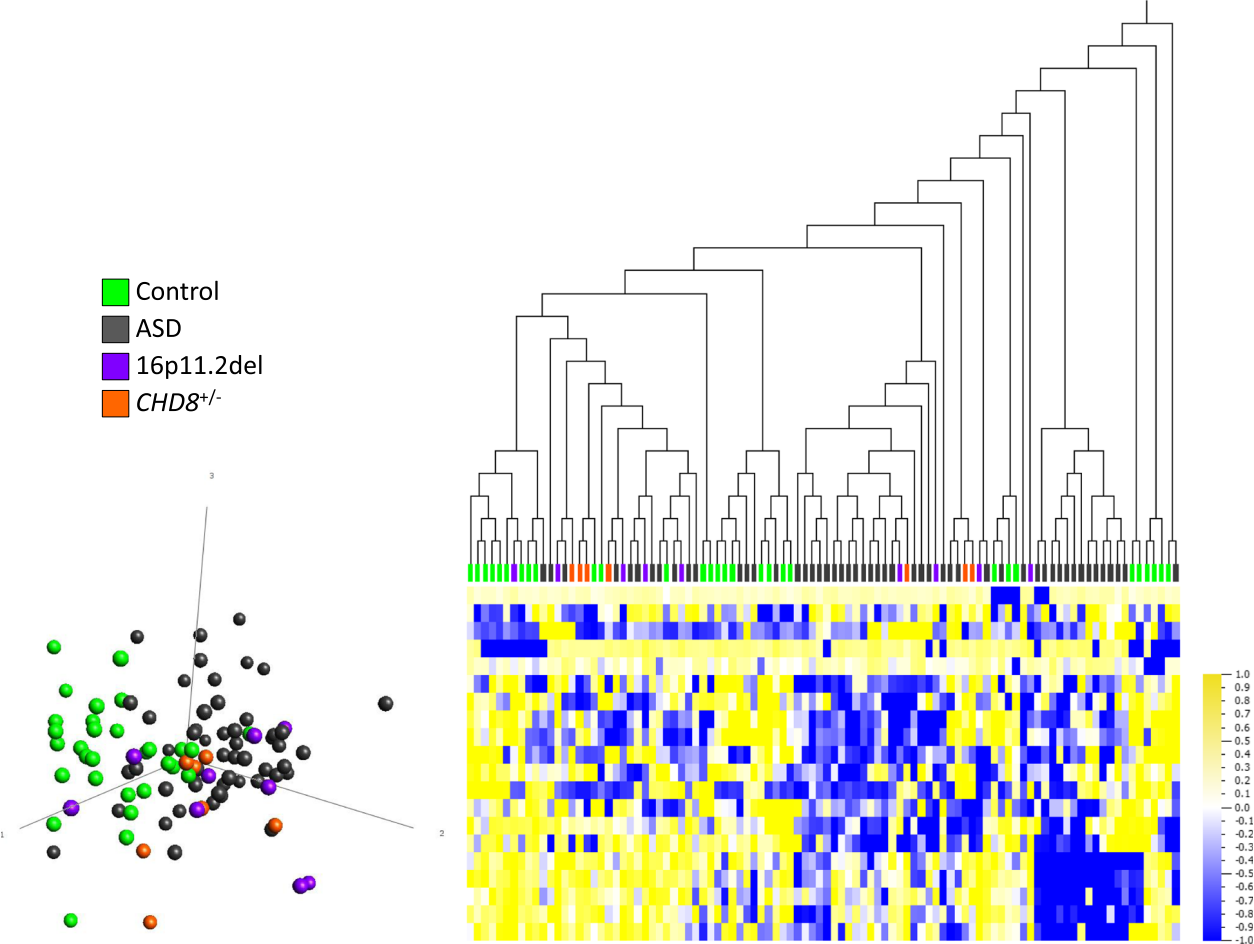
Whole blood DNAm comparison between heterogeneous ASD group (*n* = 52) and age-, sex-matched controls (*n* = 30). Following filtering by variance (40,550 sites), limma regression and Mann-Whitney *U* comparison, no CpG sites meet significance criteria of *q* ≤ 0.05. The 22 sites shown at uncorrected *p* < 0.001, |Δβ| ≥ 5% do not distinguish clearly between ASD cases and controls. Hierarchical clustering (Euclidian) and principal component analysis (PCA, first 3 principal components labeled) plot show that 16p11.2del (purple) and *CHD8*^*+/−*^ (orange) cases are mixed with both heterogeneous ASD cases (gray) and controls (green). Data are normalized for visualization (mean = 0, variance = 1). In heat map, yellow represents high methylation and blue represents low methylation

Given that the genetically homogeneous groups, 16p11.2del and *CHD8*^*+/−*^, were selected for their potential for elucidating epigenetic dysregulation, we assessed whether these groups could be distinguished epigenetically from the heterogeneous ASD group and controls. The 16p11.2del and *CHD8*^*+/−*^ cases were therefore classified at the 22 sites where putative ASD differences were detected (*p* < 0.001, |Δβ| ≥ 5%). No distinct clustering of either group was apparent, i.e., all 16p11.2del and *CHD8*^*+/−*^ cases were mixed with both ASD cases and controls (Fig. 1). These data suggested that any potential 16p11.2del- or *CHD8*^*+/−*^-specific DNAm alterations were obscured by the presence of other heterogeneous ASD cases. Therefore, we focused next on the potential discovery of specific DNAm signatures, contingent on direct comparisons of genetically homogeneous ASD-risk groups with controls.

### Identification of a 16p11.2del-specific DNAm signature

**Fig. 2:**
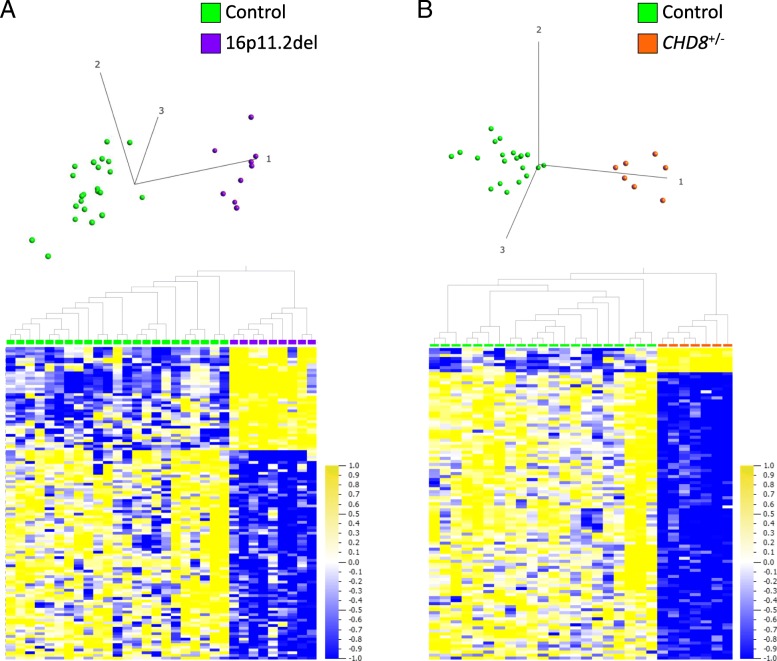
**a, b** DNAm signatures identified in whole blood of individuals with 16p11.2del (600 kb risk locus) or *CHD8*^*+/−*^. **a** Hierarchical clustering and PCA plot (first 3 principal components labeled) show that 16p11.2del training cases (purple; *n* = 9) are distinct from age-, sex-matched controls (green; *n* = 23) at the DNAm signature sites (115 CpG sites; *q* < 0.05, absolute methylation difference (|Δβ| ≥ 5%). **b** Hierarchical clustering and PCA plot (first 3 principal components labeled) show that *CHD8*^*+/****−***^ training cases (orange; *n* = 7) are distinct from age-, sex-matched controls (green; *n* = 21) at DNAm signature sites (103 CpG sites; *q* < 0.01, |Δβ| ≥ 5%) used for classification. Only a single CpG site overlaps between the 16p11.2del and *CHD8*^*+/−*^ DNAm signatures: cg25970491 (*CLTCL1*), which is hypomethylated in both *CHD8*^*+/−*^ and 16p11.2del cases relative to controls. All data are normalized for visualization (mean = 0, variance = 1). In heat map, yellow represents high methylation and blue represents low methylation

Following filtering by variance (24,009 sites remaining), comparison of the DNAm data from the training 16p11.2del group (*n* = 9; Table 1) to our age-, sex-matched controls (*n* = 23) showed that our stratification approach allowed cases to be distinguished from controls based on DNAm differences at specific genomic sites. These distinctions are demonstrated by both hierarchical clustering and PCA plot (Fig. 2a). One hundred fifteen sites (77 (67%) hypomethylated, 38 (33%) hypermethylated) distributed across the genome passed our criteria of *q* < 0.05 and |Δβ| ≥ 5% (|Δβ| range 8–23%). This set of sites constitutes the 16p11.2del DNAm signature (Additional file 2: Figure S2; Additional file 3: Table S6); none of these 115 sites are contained in the 16p11.2del region.

### Identification of a *CHD8*^*+/−*^-specific DNAm signature

We compared DNAm data from a group of individuals with known pathogenic *CHD8* variants (*n* = 7) (Table 2) to age-, sex-matched controls (*n* = 21). Following filtering by variance (87,662 sites remaining), 422 sites (392 (92.9%) hypomethylated, 30 (7.1%) hypermethylated) distributed across the genome passed our criteria of *q* < 0.05, |Δβ| ≥ 5% (|Δβ| range 5–54%) (Additional file 3: Table S7) and distinguished all *CHD8*^*+/−*^ training cases from controls. This set of sites, which constitutes the *CHD8*^*+/−*^ DNAm signature, was used for downstream biological and classification analyses. Importantly, this *CHD8*^*+/−*^ DNAm signature (422 sites) also accurately classified all *CHD8* test case sequence variants (matching in silico predictions) and classified GEO controls with > 99.3% specificity. This DNAm signature classified all but one heterogeneous ASD case as clustering with controls (data not shown), suggesting the need to increase stringency of the signature for more accurate classification.

**Fig. 3:**
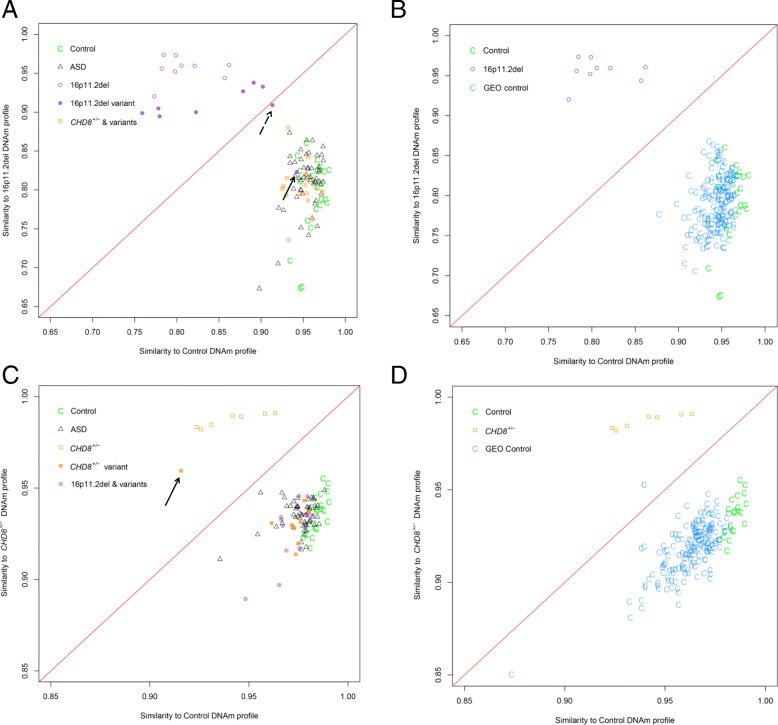
**a**–**d** Evaluating the sensitivity and specificity of the 16p11.2del and *CHD8*^*+/−*^ DNAm signatures. The classification models (details in Ref. [15]) are represented by the following classification plots: **a** The classification model based on the 16p11.2del DNAm signature sites (115 sites; *q* < 0.05, |Δβ| ≥ 5%) accurately classified *CHD8*^***+/−***^ signature cases, and independent test cases consisting of *CHD8* sequence variants (orange box with cross) and heterogeneous ASD cases (open gray triangle) as more similar to controls (green C). The model also accurately classified 7 of 9 independent 16p11.2del test cases (solid purple circle) as more similar to 16p11.2del signature cases (open purple circle), demonstrating 100% sensitivity. Two variants (arrows) correctly received negative classification scores: hatched arrow indicates a mosaic 16p11.2del case (2-0088-003), solid arrow indicates a 16p11.2del distal to and not overlapping the 600 kb typical deletion region (1-0616-003). **b** The 16p11.2del DNAm signature was tested on an independent set of compiled blood control DNAm data (*n* = 162) extracted from the Gene Expression Omnibus (GEO) Database. All GEO controls (turquoise C) were properly classified with experimental controls (green C), as distinct from 16p11.2del signature training cases (open purple circle), demonstrating 100% specificity. **c** The classification model based on the *CHD8*^*+/−*^ DNAm signature sites for classification (103 sites; *q* < 0.01, |Δβ| ≥ 5%) accurately classified 16p11.2del signature cases and independent test cases consisting of 16p11.2del variants (purple circle with cross) and heterogeneous ASD cases (gray triangle) as more similar to controls (green C). The model also accurately classified 1 of 9 independent *CHD8* test cases consisting of sequence variants (solid orange square) as more similar to *CHD8*^*+/−*^ signature training cases (open orange square) (unable to report sensitivity with one positive case, indicated by arrow). Eight variants correctly received negative classification scores (7 missense, 1 with an in-frame deletion in the last exon of *CHD8*, not predicted pathogenic). Classifications are in agreement with in silico predictions. **d** The *CHD8*^*+/−*^ DNAm signature was tested on the same GEO controls (turquoise C) as in **b**; all but one GEO control were properly classified with experimental controls (green C), as distinct from *CHD8*^*+/−*^ signature cases (open orange square), demonstrating >99.3% specificity

Therefore, we investigated whether the use of a more stringent filter of statistical significance (*q* < 0.01 and |Δβ| ≥ 5%) could be used to derive a more specific DNAm signature. Applying this filter reduced the previously derived DNAm signature to a subset of 103 CpG sites (95 (92.2%) hypomethylated, 8 (7.6%) hypermethylated) with a |Δβ| range of 5–18% (Additional file 2: Figure S3; Additional file 3: Table S7) that was used for classification purposes. Hierarchical clustering and PCA plot demonstrate that at this set of sites, cases are still distinguished clearly from controls (Fig. 2b), and that heterogeneous ASD cases and *CHD8* test sequence variants are classified with even greater precision (Fig. 3c) (discussed in greater detail in the “Sensitivity and specificity of the CHD8+/^*−*^-specific DNAm signature” section).

### Comparison of DNAm signatures with identified DMRs

Using an alternative analytic approach, we evaluated DMRs to detect regional DNAm differences and to confirm consistent patterns overlapping the significant CpG sites identified in our DNAm signatures. Significant DMRs were defined by *p* < 0.05, |Δβ| ≥ 5% and at least 3 consecutive CpG sites, of which at least one has already been identified in the 16p11.2del (115 sites) and *CHD8*^*+/−*^ (422 sites) DNAm signature CpG sites (Additional file 3: Tables S3 and S4). DMRs found in the case-control comparisons yielded 26 and 70 overlapping CpG sites with the 16p11.2del and *CHD8*^*+/−*^ DNAm signatures, respectively. Representative examples of DMR bumps that overlap DNAm signature sites are depicted in Additional file 2: Figure S4.

### The 16p11.2del and *CHD8*^*+/−*^ DNAm signatures are not confounded by blood cell type

We have taken blood cell type proportion estimates into account as a possible confounding factor by including them as covariates in the derivation of each DNAm signature. To further demonstrate that differences in cell type proportion do not contribute to the differences identified in our 16p11.2del and *CHD8*^*+/−*^ DNAm signatures, we show that all normal purified blood cell types from the study by Reinius et al., 2012 [41] (data available from GEO35069) are correctly classified as controls by both signatures (similarly applied in Ref. [14, 15]). None of the methylation profiles for any of the specific blood cell types cluster with or near the signature cases at each respective signature, indicating that cell type-specific methylation profiles are not driving the differences identified at signature sites. Therefore, our DNAm signatures are not confounded by DNAm variation associated with blood cell types (Additional file 1; Additional file 2: Figures S5 and S6).

### Sensitivity and specificity of the 16p11.2del-specific DNAm signature

We used the 16p11.2del DNAm signature to classify 9 additional 16p11.2del test case variants. For these cases, 7 of 9 received positive scores. These 7 cases receiving positive scores all carried typical 16p11.2del, clustering closely with signature cases (Fig. 3a; Table 3). Two of 9 cases received negative scores, appearing either on the diagonal border (2-0088-003) or clustering more closely with controls (1-0616-003) (Fig. 3a; Table 3). One of these two intermediate cases is known to be mosaic for the 16p11.2del (referred to as MM0088-003 in Ref. [32]), explaining its borderline position. The second case has a 200 kb deletion distal to the typical deletion region. Thus, our 16p11.2del DNAm signature demonstrates 100% sensitivity, accurately detecting all 7 variants with typical 16p11.2del. The 16p11.2del signature also accurately classified a larger set of independent GEO control subjects (*n* = 162), clustering them closely with controls, thus demonstrating 100% specificity (Fig. 3b; Additional file 3: Table S5). All *CHD8*^*+/−*^ cases were classified as negative using the 16p11.2del DNAm signature.

### Sensitivity and specificity of the *CHD8*^*+/−*^-specific DNAm signature

**Fig. 4:**
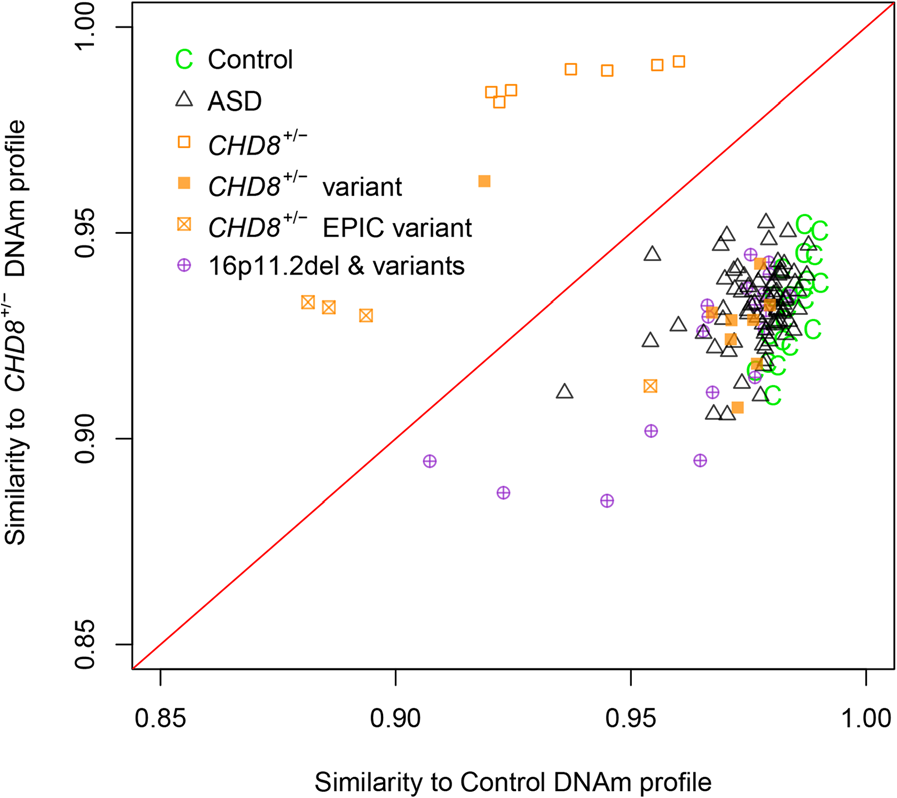
Evaluating the sensitivity of the *CHD8*^*+/−*^ DNAm signature using additional *CHD8* test cases (EPIC array). Additional independent test cases consisting of *CHD8* sequence variants (*n* = 4; orange squares with cross) were run on the EPIC array. Of the 103 sites in the 450K-derived *CHD8*^*+/−*^ DNAm classification signature, 92 sites overlapped those of the EPIC array and were thus used to classify the additional EPIC test cases. One VUS case received a negative classification score. Three of the four additional *CHD8* test cases were accurately classified as more similar to *CHD8*^*+/−*^ signature training cases (open orange square); these cases were known to have pathogenic *CHD8* variants, demonstrating 100% sensitivity. The one sample classified as benign was an inherited missense variant, similar to those 450K test cases receiving negative scores. All other cases and controls (450K) were accurately classified as in Fig. 3

We used the more stringent, 103 CpG site subset of the *CHD8*^*+/−*^ DNAm signature to classify additional *CHD8* test case sequence variants (*n* = 13: *n* = 9 samples using 450K arrays, *n* = 4 using EPIC arrays) in order to predict pathogenicity. These classifications were compared with results from in silico prediction algorithms: PolyPhen-2 (http://genetics.bwh.harvard.edu/pph2/), Mutation Assessor (http://mutationassessor.org), SIFT (http://sift-dna.org), Mutation Taster (http://www.mutationtaster.org). One test case (450K) (1-0559-003) received a positive score, classifying it as pathogenic, clustering more closely with the *CHD8*^*+/−*^ training signature cases (Fig. 3c; Table 3). This case, which has a de novo heterozygous frameshift nonsense mutation in *CHD8*, was also classified as pathogenic by Mutation Taster, the only predictor available due to the nature of the variant. All 8 of the other *CHD8* test case variants run on the 450K (including VUS) received negative scores, classifying as benign and clustering with controls (Fig. 3c; Table 3). For these 8 *CHD8* sequence variants, 5 were missense/synonymous mutations inherited from an unaffected parent (inheritance unknown for 3 samples) and one was an in-frame deletion in the last exon. At least three out of four in silico analyses supported the benign designation of these *CHD8* variants. All but one GEO control sample were classified correctly, clustering with controls, thus demonstrating > 99.3% specificity (Fig. 3d; Additional file 3: Table S5). Classification of the 4 *CHD8* test cases run on the EPIC array was performed using only the overlapping sites from the 450K DNAm signature (92/103 sites) (Fig. 4). These 92 sites classified 3 test cases as pathogenic, clustering more closely with signature cases, and one VUS as benign, clustering with controls. The 3 test cases receiving positive scores were all known to have pathogenic *CHD8*^*+/−*^ variants. The one sample classified as benign was an inherited missense variant, similar to those 450K test cases receiving negative scores. All 16p11.2del cases were classified as negative using the *CHD8*^*+/−*^ DNAm signature.

All the heterogeneous ASD cases (*n* = 52) received negative scores when classified with the 16p11.2del or *CHD8*^*+/−*^ DNAm signature used for classification, and all clustered with controls (Fig. 3a, c).

### Genomic distribution of DNAm signature CpG sites

CpG sites in the 16p11.2del DNAm signature (115 sites) were found to be significantly enriched in CpG islands, shelves, shores, promoters, and DNase hypersensitivity sites (DHS), and depleted in shelves (*p* < 0.05) (Additional file 2: Figure S7A). Sites in the 422 probe *CHD8*^*+/−*^ DNAm signature were enriched in islands, shores, particularly the “south” shores (S_Shore) directly downstream of CpG islands, differentially methylated regions (DMRs) and DHS, and depleted in shelves and reprogramming-specific DMRs (RDMRs) (*p* < 0.05); whereas the 103 site *CHD8*^*+/−*^ DNAm signature used for classification was enriched in shores, particularly in S_Shore, promoters, and DMRs (Additional file 2: Figure S7B, C).

### Biological significance of the 16p11.2del and *CHD8*^*+/−*^ signatures

**Fig. 5:**
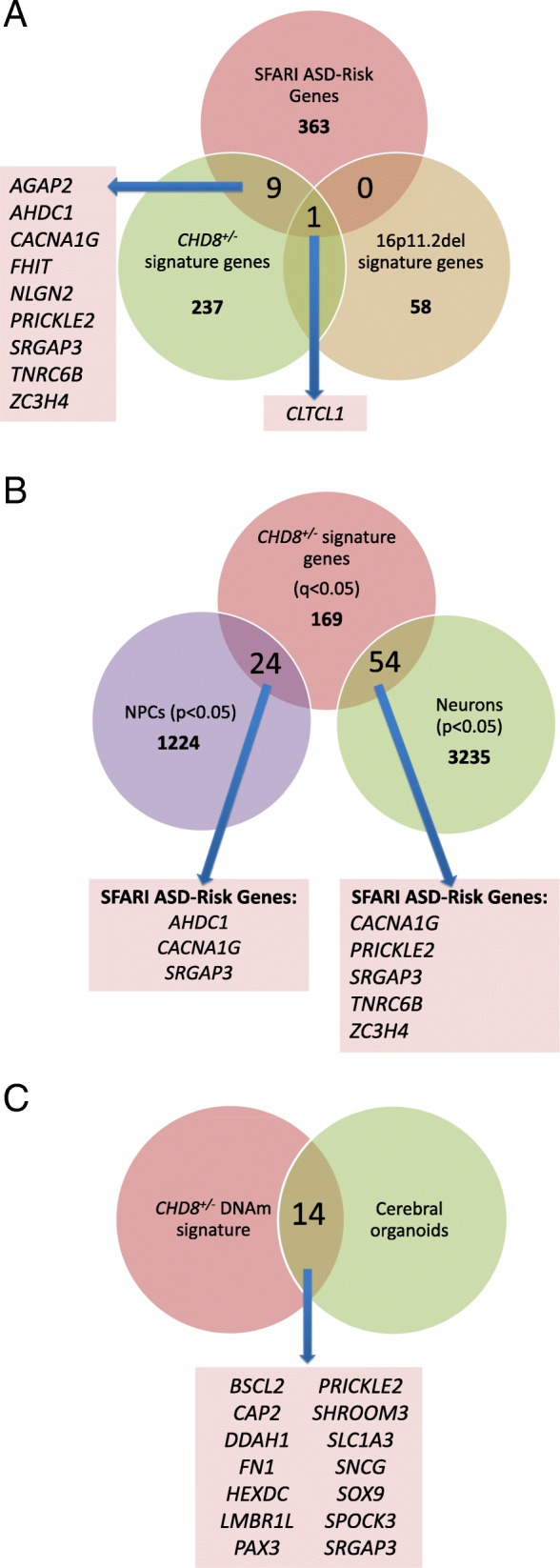
Biological and cross-tissue functional significance of differentially methylated genes. **a** Differentially methylated genes associated with the 16p11.2del and *CHD8*^*+/−*^ DNAm signatures (*q* < 0.05, |Δβ| ≥ 5%) and the DNAm signature overlap with known ASD-risk genes (SFARI Gene). A single SFARI gene, clathrin heavy chain like 1 (*CLTCL1*), is significantly hypomethylated in both groups. An independent set of 9 SFARI genes are differentially methylated in the *CHD8*^*+/−*^ DNAm signature. **b** Our findings are further corroborated by an independent study (48) showing that *CHD8*^*+/−*^ human iPSC-derived neuronal precursor cells (NPCs) and differentiated neurons result in differentially expressed genes that overlap with some of the differentially methylated genes in *CHD8*^*+/−*^ DNAm signature, including known ASD-risk genes (SFARI Gene), and **c** another study (49) showing that cerebral organoids derived from the iPSCs in **b** also have differentially expressed genes that overlap our *CHD8*^*+/−*^ DNAm signature.

Using GREAT 3.0.0, we identified molecular and biological processes potentially relevant to the phenotype of individuals with 16p11.2del or *CHD8*^*+/−*^ using the differentially methylated CpGs from each respective DNAm signature. When we examined the 16p11.2del signature (115 sites), 3 of 5 GO Biological Process terms with significant enrichment (hypergeometric FDR *q*-value < 0.05) appeared in categories related to immune function (Additional file 3: Table S8). When we examined the *CHD8*^*+/−*^ DNAm signature (422 sites), > 20 terms passed with a more stringent hypergeometric FDR *q*-value < 0.05 (Additional file 3: Table S9). The top 20 terms showed enrichment not only in immune processes, but also in the regulation of inhibitory postsynaptic membrane potential and central nervous system development. Differentially methylated genes specific to 16p11.2del and *CHD8*^*+/−*^ signatures overlap with 1 and 10 SFARI Gene ASD-risk genes, respectively (Fig. 5a). The clathrin, heavy chain-like 1 *(CLTCL1)* gene was hypomethylated in both signatures.

### Cross-tissue functional relevance of the *CHD8*^*+/−*^ signature

To examine whether DNAm changes downstream of sequence variants in *CHD8* in blood are relevant to neuronal function, we compared the overlap between genes in the *CHD8*^*+/−*^ DNAm signature to differentially expressed genes (DEGs) in human *CHD8*^*+/−*^ iPSC-derived NPCs and neurons (48). Differentially expressed genes in NPCs and neurons overlapped 24 (hypergeometric *p* value < 0.001) and 54 (hypergeometric *p* value < 0.001) of our 247 differentially methylated genes in the DNAm signature (*q* < 0.05, |Δβ| ≥ 5%), respectively (Fig. 5b). Furthermore, 6 of these overlapping genes also overlapped SFARI ASD-risk genes.

More recently, Wang et al. [49] further derived cerebral organoids from their original *CHD8*^*+/−*^ iPSCs. These organoids are composed of GABAergic/glutamatergic neurons, and radial glia progenitor cells with gene expression profiles that resemble first-trimester telencephalon [49–52]. The *CHD8*^*+/−*^ DNAm signature overlapped 14 DEGs found in the *CHD8*^*+/−*^ cerebral organoids (Fig. 5c); 6 of these overlapped DEGs found in both organoids and neurons, and 5 of these overlapped DEGs found in both organoids and NPCs.

### Validation of differentially methylated CpG sites with targeted pyrosequencing

Specific CpG sites were selected from the DNAm signatures for validation by targeted pyrosequencing (Additional file 2: Figure S4). These sites overlapped a significant DMR that contained at least two sites within a single gene. For 16p112del cases, the following CpG sites were validated: cg00108944, cg23588049 in *GLIPR1L2*, cg25983544, and cg06377543 in *PSMA8*. In the *CHD8*^*+/−*^ group, the following CpG sites were validated: cg09819656, cg15089111 in *NPAS3*, cg27206976, and cg04089788 in *PLXNB2*. In the 16p11.2del sites, an average gain of methylation (GOM) by 14.5% and 13% at cg00108944 and cg23588049 in *GLIPR1L2*, respectively, and loss of methylation (LOM) by 7.4% and 10.5% at cg25983544 and cg06377543 in *PSMA8*, respectively were confirmed by our pyrosequencing analysis (*p* < 0.005). In the *CHD8*^*+/−*^ sites, an average GOM by 17% and 18.8% at cg09819656 and cg15089111 in *NPAS3*, respectively, and LOM by 11.4% and 18.9% at cg27206976 and cg04089788 in *PLXNB2*, respectively, were confirmed (*p* < 0.005). The correlation between methylation *β* values from the 450K array and % methylation values obtained through pyrosequencing were examined and were found to be highly concordant (*r* = 0.8524) (Additional file 2: Figure S8).

## Discussion

One of the greatest challenges of epigenetic research in ASD is the degree of etiologic and phenotypic heterogeneity. This study uniquely demonstrates that investigating homogeneous subgroups of individuals with genomic alterations at ASD-risk loci involving epigenes enables the identification of distinct functionally and biologically relevant DNAm signatures. Previously, evaluations of DNAm in individuals with ASD have focused on heterogeneous ASD cases in different tissue types using a variety of different methods. These studies of modest sample sizes (*n* < 50) have identified few, mostly non-overlapping DNAm alterations of small magnitude [24, 26–29, 31].

Heterogeneous ASD cases, when compared with controls, did not identify significant (i.e., corrected for multiple testing) differentially methylated sites, paralleling the aforementioned outcomes of DNAm studies in ASD [24, 26–31]. We expect that the identification of significant epigenetic differences in such a mixed cohort will require large numbers (> 1000) of ASD cases. We therefore undertook a novel alternative approach to identifying etiologically relevant epigenetic alterations in ASD. In contrast to the current literature, we sought to reduce genetic and epigenetic heterogeneity by focusing analyses on groups of individuals with genomic alterations involving epigenes that confer an increased risk for ASD. We show that a relatively small number (< 10) of genetically homogeneous cases can be clearly distinguished from both a heterogeneous ASD group and neurotypical controls based on DNAm. In the future, these findings will need to be replicated using larger cohorts of cases with a wider range of risk-associated variants. Given the expected genomic heterogeneity for ASD, we did not expect that our heterogeneous cohort of ASD patients would contain any individuals with either 16p11.2del or *CHD8*^*+/−*^ pathogenic variants as each of these individually account for < 1% of all ASD cases. We detected consistent patterns of DNAm alterations in the 16p11.2del and *CHD8*^*+/−*^ blood DNAm signatures by identifying overlapping DMRs. In addition, we validated the top differentially methylated CpG sites in the DNAm signatures by targeted bisulfite pyrosequencing. We demonstrate that our DNAm signatures are robust, overcoming potential confounding factors such as sex, age, blood cell type proportions, and technical variation, illustrated by our classification of a large number of independently assessed GEO control blood DNAm samples. Some additional confounding factors to consider include ethnicity and environmental exposures such as medications, prenatal vitamins, and smoking. Data for these parameters were not collected consistently across our cases or controls, precluding us from incorporating these factors into our models. However, given that our signatures yielded 100% sensitivity and > 99% specificity for both signatures, despite a wide variety of potentially confounding variables, we expect that our signatures are robust enough to overcome these additional confounders. Further, we were able to demonstrate cross-platform applicability of our *CHD8*^*+/−*^ DNAm signature derived on the 450K array. Using this signature, we accurately classified 4 *CHD8* sequence variant test cases that were run on the EPIC array.

It is important to recognize that DNAm signatures are dynamic; their stability and robustness depend on the size and composition of the group of signature cases used to derive the signature. DNAm signatures can be optimized in various ways depending on the specific purposes for which they are to be utilized. For example, one signature may provide more information for the examination of underlying biological pathways of disease, whereas classification of sequence variants in a clinical diagnostic setting may require a more refined signature to optimize sensitivity and specificity. DNAm signatures can be further optimized to reduce type I error inflation, which may confound the performance of a signature in a classification model (see Additional file 1). Although we were only able to test our signatures on a small group of test cases, we were able to demonstrate 100% sensitivity. Refining a signature for clinical diagnostic application will require a larger discovery cohort with a wider range of variant types and genomic locations (e.g., alterations across different functional domains) to improve classification performance and to better understand their impact on pathogenicity and functional consequences.

The identification of the 16p11.2del and *CHD8*^*+/−*^ DNAm signatures highlights both the scientific relevance and the potential for clinical utility. Highly sensitive and specific genome-wide DNAm signatures have the potential to be used to identify individuals with pathogenic variants among groups of heterogeneous ASD cases, an approach currently in development for molecular diagnostics. As well, our DNAm signatures are able to facilitate the interpretation of VUS of the respective CNV or epigene variants detected using sequencing/array-based diagnostics. This particular application underscores the potential clinical utility of this molecular classification system beyond simply genotyping using NGS technologies. Given that there are already gene-specific panels available to test for variants in ASD-risk populations, we propose that in the future, a parallel panel could be used as a complementary second-tier diagnostic test that would employ various ASD risk-specific DNAm signatures. Finally, differentially methylated genes that constitute the downstream genomic targets of ASD-associated CNVs and sequence variants in epigenes elucidate the biological and molecular pathophysiology of ASD and identify potential therapeutic targets. Expanding this work in the future could also lead to the identification of biomarkers for earlier prediction of neurodevelopmental outcomes, which would be of tremendous value given the variable penetrance of ASD with each ASD-risk locus. Although our current study does not have sufficient statistical power to accomplish this, the future study of large numbers of cases with 16p11.2del and *CHD8* sequence variants in conjunction with deep clinical phenotyping may enable the identification of more refined epigenotype-phenotype correlations. Further investigation will also be required to better understand the impact of altered methylation on downstream targets for each DNAm signature in a cell type-, tissue-, and developmental time-specific manner.

Genes and pathways relevant to the clinical phenotypes of individuals with 16p11.2del or *CHD8*^*+/−*^ are elucidated by their respective DNAm signatures. Interestingly, the single gene, *CLTCL1*, found to be similarly hypomethylated in both the 16p11.2del and *CHD8*^*+/−*^ signatures, is found in the 22q11.2 deletion syndrome (22qDS; OMIM# 611867) deletion region. 22qDS is associated with a range of clinical phenotypes, but notably, several neurodevelopmental (ASD, ADHD), and neuropsychiatric (schizophrenia) outcomes [53]. In the 16p11.2del signature, multiple sites in GLI pathogenesis-related 1 like 2 (*GLIPR1L2;* 8 hypermethylated sites, |Δβ| > 10%) and proteasome subunit alpha 8 (*PSMA8*; 3 hypomethylated sites, |Δβ| > 8%) were found to be significantly differentially methylated. *GLIPR1L2* and *PSMA8* have not previously been associated with ASD, but encode proteins that function in immunity and histone degradation, respectively. The *CHD8* gene is known to negatively regulate beta catenin (CTNNB1) and *WNT* pathways, both important for development and growth, through the direct interaction between CHD8 and CTNNB1. Our study extends the current knowledge of downstream targets regulated by *CHD8* through its role as a chromatin remodeler. The genes with the greatest differential DNAm in our *CHD8*^*+/−*^ signature highlight pathways that are relevant to the pathophysiology of ASD. These include Plexin B2 (*PLXNB2;* 4 hypomethylated sites, |Δβ| > 12%) and neuronal PAS domain protein 3 (*NPAS3*; 2 hypermethylated sites, |Δβ| > 15%), both of which are expressed in the brain during embryonic development [54, 55]. *PLXNB2* encodes a transmembrane receptor protein that participates in axon guidance and is required for normal embryonic development/differentiation and migration of neuronal cells during brain corticogenesis [54, 56]. *PLXNB2* is deleted in the Phelan-McDermid, 22q13.3 deletion syndrome region, which is associated with dysmorphic features, neurologic deficits and ASD. This overlap could suggest that epigenetic dysregulation of shared biological pathways across disorders is reflected in overlapping clinical features. This has been demonstrated in the case of CHARGE and Kabuki syndromes, two clinically distinct syndromes sharing a subset of phenotypes [14]; each syndrome has a distinct DNAm signature, each of which also includes some overlapping sites affecting genes involved in pathways relevant to specific concordant syndromic features. Mouse models with *Plxnb2* deletions are perinatal lethal and have abnormal cerebellar development, reflecting the critical role of the gene in neurodevelopment [56]. *NPAS3*, expressed by GABAergic interneurons, has been shown to be an important transcription factor during neurogenesis; chromosomal aberrations encompassing this gene are associated with schizophrenia and mental retardation [55, 57]. The finding that immune-related genes are enriched in both the 16p11.2del and *CHD8*^*+/−*^ signatures supports recent data highlighting contributions of altered immune function to ASD pathophysiology [58–60]. Individuals with 16p11.2del or *CHD8*^*+/−*^ do not demonstrate consistent immune phenotypes; however, the dysregulation of immune genes could mediate changes in early embryogenesis that may affect neurogenesis and subsequently ASD risk. Immune dysregulation likely only contributes to the pathophysiology of disease for a subset of individuals with ASD, rather than all individuals. Importantly, the identification of these pathways could help to identify targets for potential drug therapies and facilitate more individualized interventions.

We searched for overlap between differentially methylated sites in our 16p11.2del and *CHD8*^*+/−*^ blood DNAm signatures and previous DNAm studies of ASD. Two differentially methylated genes (*DNPEP*, *RAD50*) in the *CHD8*^*+/−*^ signature overlapped with the single 450K array study of DNAm in the blood of individuals with ASD [29]. Differentially methylated genes identified in the brain regions Brodmann areas (BA) 10 and 24 of the cerebral cortex (27) overlap a number of differentially methylated DNAm signatures genes in our study: 16 genes in the 16p11.2del signature, 51 genes in the *CHD8*^*+/−*^ signature in BA10 and 22 genes in the 16p11.2del signature, and 82 genes in the *CHD8*^*+/−*^ signature in BA24. A more recent study also examining DNAm in the postmortem brain (prefrontal and temporal cortices, cerebellum and cross-cortical analysis) in individuals with idiopathic ASD identified region-specific significant differentially methylated sites [30]. Although none of our signature sites overlapped those identified in Wong et al. [30], both studies identified pathway enrichment in immune and neuronal processes, which were found using two different analytical pipelines (GREAT in our study, weighted gene co-methylation network analysis in Ref. [30]). These data suggest that certain DNAm alterations found in blood are likely to be reflected in the brain or in genes of related biological pathways, which is of particular relevance for research in neurodevelopmental disorders (NDDs). Although ASD is an NDD with heterogeneous etiology(ies) and phenotypes, where the brain is undoubtedly an important organ to study, the pathophysiology of ASD is still unclear, affecting multiple systems (e.g., gastrointestinal) and pathways (e.g., metabolic, immune). DNAm in blood also reflects environmental exposures (e.g., gestational alcohol exposure). It is important to distinguish between the different potential utilities of DNAm signatures. From a biomarker perspective, whether genes identified by a DNAm signature in blood can be causally linked to the disease phenotype is less important than identifying DNAm marks that predict the phenotype in a stable, sensitive, and specific manner. From a biological perspective, an increasing number of studies do in fact demonstrate an intersection between DNAm patterns in the blood and brain using similar genome-wide assessment platforms [61–63], which may help to elucidate underlying pathophysiological mechanisms. Our findings in blood are further corroborated by an independent study [48, 49] showing that CRISPR/Cas9-mediated creation of *CHD8*^*+/−*^ human iPSC-derived NPCs, differentiated neurons, and cerebral organoids results in differentially expressed genes that overlap with some of the differentially methylated genes in our *CHD8*^*+/−*^ DNAm signature (Fig. 5b, c), including known ASD-risk genes (SFARI Gene).

It is possible that epigenetic marks other than DNAm at CpG sites, such as 5-hydroxymethylcytosine (5-hmC), may also demonstrate differences between individuals with ASD and neurotypical controls. 5-hmC is abundant in the brain and not in the blood [64, 65] and is yet to be explored with respect to ASD in humans, although mouse models of ASD have demonstrated genome-wide differences [66]. A potential role for altered histone acetylation in ASD has been demonstrated in a recent human brain histone acetylome study [67]. Animal studies have more extensively investigated the effects of in utero exposure to a histone deacetylase inhibitor, valproic acid, on autistic-like behavioral and cognitive outcomes in offspring [68–70]. Further research exploring downstream molecular functional studies such as gene and protein expression are also necessary to fully understand the functional consequences of either the ASD-associated CNV or gene variants of interest in our study. Nevertheless, our DNAm signatures represent novel DNA biomarkers in an easily accessible tissue (blood) that are more stable than histone marks and present novel clinical diagnostic applications. Our DNAm signatures include alterations in genes that may be important for understanding the underlying pathophysiology of ASD and demonstrate parallel relevance in gene expression profiles within cell types representative of the brain.

## Conclusions

In summary, we present a novel approach that enhances the detection of epigenetic dysregulation in ASD etiology. Our successful identification of highly sensitive and specific CNV/gene mutation-specific DNAm signatures provides significant opportunities for clinical impact, offering more precise ASD subgroup molecular classification with the potential for developing diagnostic and predictive biomarkers. Our investigations provide a more comprehensive understanding of the molecular landscape underlying genetically homogeneous groups of ASD, illustrating the convergence of epigenetic and genetic mechanisms. These findings will also help to further elucidate the molecular pathophysiology of ASD and facilitate the identification of novel therapeutic targets to support precision therapies for individuals with ASD.

## Additional files

Additional file 1: Supplementary information. (DOCX 18 kb)

Additional file 2: **Figure S1**. Data analysis flowchart. Outline of the training cohorts and analysis pipeline used to derive DNAm signatures and test cohorts (independent samples), on which the DNAm signatures were tested. **Figure S2**. Identification of differentially methylated CpG sites when comparing 16p11.2del signature cases with age-, sex-matched controls. Significant sites for the DNAm signature for 16p11.2del represented by the overlap from *limma* regression and Mann Whitney U analysis are shown. Each volcano plot represents the distribution of significant sites at various statistical parameters (*y*-axis is negative log q-value, Benjamini-Hochberg corrected) and Δβ differences (*x*-axis is the average difference in DNAm between 16p11.2del and controls). Red horizontal line represents *q* < 0.05, red vertical lines represent |Δβ| ≥ 5%. **Figure S3**. Identification of differentially methylated CpG sites when comparing *CHD8*^*+/−*^ signature cases with age-, sex-matched controls. Significant sites for the DNAm signature for *CHD8*^*+/−*^ represented by the overlap from limma regression and Mann Whitney *U* analysis are shown. Each volcano plot represents the distribution of significant sites at various statistical parameters (*y*-axis is negative log q-value, Benjamini-Hochberg corrected) and Δβ differences (*x*-axis is the average difference in DNAm between *CHD8*^*+/−*^ and controls). Red horizontal line represents *q* < 0.05, red vertical lines represent |Δβ| ≥ 5%. **Figure S4**. Targeted sodium bisulfite pyrosequencing of selected 16p11.2del and *CHD8*^*+/−*^ DNAm signature CpG sites overlapping differentially methylated regions (DMRs). Specific CpG sites from our DNAm signatures that overlapped DMRs found using bump hunting were selected for targeted sodium bisulfite pyrosequencing validation in signature cases and age-, sex-matched controls. In the 16p11.2del group, the following CpG sites were validated: A) cg00108944 and cg23588049 in *GLIPR1L2* showing a gain of methylation, B) cg25983544 and cg06377543 in *PSMA8* showing a loss of methylation. In the *CHD8*^*+/−*^ group, the following CpG sites were validated: C) cg09819656 and cg15089111 in *NPAS3* showing a gain of methylation, D) cg27206976 and cg04089788 in *PLXNB2* showing a loss of methylation. Upper graphs in each panel depict mean methylation (y-axis) over each DMR (x-axis, position of CpG sites along the chromosome) for the 16p11.2del (purple), *CHD8*^*+/−*^ (orange) and control (green) groups. Box plots represent median methylation at the specific CpG sites indicated with min/max whiskers. **Figure S5**. Correlation of pyrosequencing and array data. We demonstrate that there is high correlation (*r* = 0.8524) between the bisulfite pyrosequencing validation % methylation and array methylation beta (β) values. Data include all samples run at all 8 of the specific CpG sites assayed for validation. **Figure S6**. Comparison of the 16p11.2del DNAm signature with blood cell type composition. Blood cell type DNAm data was extracted from Reinius et al. [41] (GEO series GSE35069) representing whole blood, peripheral blood mononuclear cells, granulocytes and isolated cell populations (CD4+ T cells, CD8+ T cells, CD56+ NK cells, CD19+ B cells, CD14+ monocytes) from 6 different control individuals. Blood cell type data was combined with our signature cases and control data using only the CpGs from the 16p11.2del-specific DNAm signature. The PCA plot demonstrates that the 16p11.2del signature is independent of blood cell type composition where signature cases remain separated from controls cases (whole blood and purified blood cell types). Abbreviations: PBMC peripheral blood mononuclear cells; WB whole blood. **Figure S7**. Comparison of the *CHD8*^*+/−*^ DNAm signature with blood cell type composition. Blood cell type DNAm data was extracted from Reinius et al. (41) (GEO series GSE35069) representing whole blood, peripheral blood mononuclear cells, granulocytes and isolated cell populations (CD4+ T cells, CD8+ T cells, CD56+ NK cells, CD19+ B cells, CD14+ monocytes) from 6 different control individuals. Blood cell type data was combined with our signature cases and control data using only the CpGs from the *CHD8*^*+/−*^-specific DNAm signature. The PCA plot demonstrates that the *CHD8*^*+/−*^ signature is independent of blood cell type composition where signature cases remain separated from controls cases (whole blood and purified blood cell types). Abbreviations: PBMC peripheral blood mononuclear cells; WB whole blood. **Figure S8**. Genomic distribution of DNAm signature CpG sites. Bar charts representing the genomic distribution of CpG sites in the 16p11.2del (A) and *CHD8*^*+/−*^-specific DNAm signatures (B, C [classification signature]), according to Illumina 450K array annotations. In mammalian cells, DNAm occurs predominantly on cytosines located in CpG dinucleotides. CpG islands refer to regions that are especially CpG-rich. Specific locations of CpGs relative to the CpG island can be denoted as “shores” and “shelves”. A shore is 0–2 kb from a CpG island, a shelf is 2–4 kb from a CpG island. N refers to regions upstream (5′) and S refers to regions downstream (3′) of a CpG island. We compared the distribution of the CpGs between each respective variance filtered dataset with each signature training case group for the regulatory feature group, relation to CpG island and other functional categories such as overlapping enhancer region, DNase hypersensitive sites (DHS) and type of differentially methylated regions (Reprogrammed DMR (RDMR), cancer-specific DMR (cDMR), other DMR) in addition to relation to RefSeq group annotation. N refers to north and S refers to south. Asterisk denotes significant enrichment or depletion by hypergeometric test comparison (*p* < 0.05). **Figure S9**. The quantile-quantile plot showing the log-transformed distributions of limma regression p-values associated with the DNAm differences in *CHD8*^*+/−*^ cases compared to matching controls. The results are shown before (left) and after (right) correction by the BACON method, with inflation patterns evident in both cases. **Figure S10**. The quantile-quantile plot showing the log-transformed distributions of limma regression p-values associated with the DNAm differences in 16p11.2del cases compared to matching controls. The results are shown before (left) and after (right) correction by the BACON method, with inflation patterns evident in both cases. (PPTX 2477 kb)

Additional file 3: **Table S1**. Demographic information for heterogeneous ASD cases and age- and sex-matched neurotypical controls. **Table S2**. Number of probes removed and remaining for analysis following quality control. **Table S3**. List of overlapping DMRs for 16p11.2del. **Table S4**. List of overlapping DMRs for *CHD8*^*+/−*^*.* ***T*able S5**. List of samples extracted from GEO database and respective 16p11.2del and *CHD8*^*+/−*^ signature scores for specificity analysis. All sample data are from blood and from individuals with known ages < 50 years old. **Table S6**. List of significant (*q* < 0.05, |Δβ| ≥ 5%) sites in 16p11.2del DNAm signature. **Table S7**. List of significant (*q* < 0.05, |Δβ| ≥ 5%) sites in *CHD8*^*+/−*^ DNAm signature. **Table S8**. GO Biological Process enrichment terms following GREAT enrichment analysis of differentially methylated genes in 16p11.2del DNAm signature. **Table S9**. GO Biological Process enrichment terms following GREAT enrichment analysis of differentially methylated genes in *CHD8*^*+/−*^ DNAm signature. (XLSX 156 kb)

## Data Availability

The datasets used for the current study are available from the corresponding author on reasonable request.

## Abbreviations

16p11.2del: 16p11.2 deletion
ASD: Autism spectrum disorder
BA: Brodmann area
cDMR: Cancer-specific DMRs
CHARGE: Coloboma of the eye, Heart defects, Atresia of the choanae, Retardation of growth and development, and Ear abnormalities and deafness syndrome
CHD7: Chromodomain helicase DNA-binding protein 7 gene
CHD8: Chromodomain helicase DNA-binding protein 8 gene
CLTCL1: Clathrin, heavy chain-like 1 gene
CNV: Copy number variant
CTNNB1: Beta catenin
DEG: Differentially expressed gene
DHS: DNase hypersensitivity site
DMR: Differentially methylated region
DNAm: DNA methylation
GEO: Gene Expression Omnibus database
GLIPR1L2: GLI pathogenesis-related 1 like 2 gene
HIRIP3: HIRA*-*interacting protein 3 gene
iPSC: Induced pluripotent stem cell
NPAS3: Neuronal PAS domain protein 3 gene
NPC: Neuronal precursor cells
NSD1: Nuclear receptor binding SET domain protein 1 gene
PCA: Principal component analysis
PLXNB2: Plexin B2 gene
PSMA8: Proteasome subunit alpha 8 gene
QOE: Qlucore Omics Explorer
RDMR: Reprogramming-specific DMR
VUS: Variant of unknown significance
WB: Whole blood
Δβ: Effect size of differential DNA methylation
