# Supplementary material for: Functional DNA methylation signatures for autism spectrum disorder genomic risk loci: 16p11.2 deletions and CHD8 variants
Source: Clin Epigenetics. 2019 Jul 16;11:103. doi: 10.1186/s13148-019-0684-3 (PMC6636171; doi:10.1186/s13148-019-0684-3)
Supplement: Supplementary file 1 — Supplementary information. (DOCX 18 kb) [file 13148_2019_684_MOESM1_ESM.docx]

**SUPPLEMENTARY INFORMATION**

**Blood cell type analysis**

We predicted the proportion of CD4+ T cells, CD8+ T cells, NK cells, B cells, monocytes and granulocytes using the *minfi* package implemented in R Studio (v.3.4.1) for each sample. Results were tested for normality to determine if groups could be compared using a t-test (normally distributed) or a Mann-Whitney test (non-normally distributed) with significance set at p < 0.05.

To further demonstrate that blood cell type proportion is not confounding our DNAm signatures, we compared our data to DNAm data for 6 controls with 8 sorted blood cell subtypes each, available from GEO (series GSE35069) (41). The dataset includes control DNAm data for whole blood (WB), peripheral blood mononuclear cells (PBMCs), granulocytes and isolated cell populations (CD4+ T cells, CD8+ T cells, CD56+ NK cells, CD19+ B cells, CD14+ monocytes). This data was combined with our training signature case and control data and analyzed at each respective classification signature using PCA plots to determine whether or not the signatures were able to independently distinguish cases from controls, i.e., not be confounded by individual differences in blood cell type proportion (Supplementary Figures 5-6), as previously applied in Refs. 14, 15.

**DNAm Validation by Targeted Sodium Bisulfite Pyrosequencing**

Sequences of primers used for validation of selected differentially methylated CpG sites from the 16p11.2del and *CHD8^+/−^* DNAm signatures (Supplementary Figures 4,8):

*GLIPR1L2:* cg00108944, cg23588049 (146 bp)

GLIPR1L2F GGGAGGAGGGATAAGGTTTGT

GLIPR1L2R AATAAAAACTAAACCCTCCACTCC

GLIPR1L2S ATTGGTAGGTTATTGGG

*PSMA8:* cg25983544, cg06377543 (126 bp)

PSMA8F GAGTGTAGTGATATTATTTTTGGGGTAA

PSMA8R ACCTTTATCACCCAAACAATACC

PSMA8S CACCCAAACAATACCT

*NPAS3:* cg09819656, cg15089111 (294 bp)

NPAS3F AGGTGATTTTGGTTATGTAGAGTAATTT

NPAS3R CCTCCTTACCCTCCAAAATCTATACC

NPAS3S TTGGTTATGTAGAGTAATTTG

*PLXNB2:* cg27206976 (62 bp)

PLXNB2F GGGGAGGGGTTGTGTTAT

PLXNB2R ACCTATATAAAACCCCCTAAACTATTCC

PLXNB2S AGGGGTTGTGTTATT

*PLXNB2:* cg04089788 (190 bp)

PLXNB2F GGTTTTTTTAGGTTTAGTTGTTAATTATGT

PLXNB2R CAAACCTCCACATCAATCTAT

PLXNB2S GATTTTGTTAGGAGTAGTTTT

**Addressing Type I Error Inflation**

Our analysis used regression modeling using R package *limma*, which accounted for confounders such as sex, age, batch and estimated blood cell type composition in each sample. To explore possible influence of additional confounders on our results, we examined the patterns of the p-values resulting from *limma* regression modeling. First, we converted the set of regression p-values into the quantiles of chi-square distribution. Then we calculated the genomic inflation factor λ, which is commonly used in genome-wide association studies (GWAS) and is defined as the ratio between the median of the observed chi-square values and the median value of the theoretical chi-square distribution with one degree of freedom. Furthermore, in epigenome-wide studies like ours (as opposed to GWAS) the inflation factor corresponds to the square root √λ rather than λ itself, due to the variance structure of continuously-valued DNAm outcomes (van Iterson et al. 2017). This resulted in the estimated inflation values √λ = 1.20 for the *CHD8^+/−^* dataset and √λ = 1.26 for the 16p11.2del dataset.

Next, we attempted to mitigate the inflation by applying a Bayesian correction method BACON (van Iterson et al. 2017), available as an R/Bioconductor package *bacon*. This method is designed specifically to account for unobserved covariates in epigenetic datasets such as the HumanMethylation450 microarray data. BACON takes as input the quantiles of standard normal distribution (z-scores), hence we transformed the set of *limma* results into the corresponding z-scores, while assigning either positive or negative sign depending on the direction of the test statistic in each CpG (i.e., positive z-scores for DNAm gain in cases compared to controls, negative for DNAm loss). The quantile-quantile plots (QQ plots) before and after BACON correction for each of the two datasets show that the general inflationary patterns persist, although reduced, but the effect of the correction was modest (Supplementary Figures 9 and 10). There was only a partial reduction in the estimated √λ inflation values: from 1.20 to 1.16 in the *CHD8^+/−^* dataset and from 1.26 to 1.05 in the 16p11.2del dataset. These results suggest that the persistence of the inflation patterns may be due to factors other than the unobserved covariates, which BACON could not eliminate.

To explore this question further, we estimated directly the presence of unobserved covariates, and whether they may contribute to the apparent differences between cases and controls (leading to Type I errors or false positives). For this we applied the Surrogate Variable Analysis (Leek et al. 2012) available via the R/Bioconductor package *sva*. This method is designed to discover structural patterns and artifacts (the so-called surrogate variables) in microarray data while preserving the differences between the main study cohorts. The number of surrogate variables was estimated using the default Buja-Eyuboglu method (option “be” in the *sva*) while indicating the sample group (i.e., cases vs. controls) as the main variable and accounting for the same confounders as previously in *limma* regression modeling. The sva detected n = 7 surrogate variables in the *CHD8^+/−^* dataset and n = 9 variables in the 16p11.2del dataset, which represent unobserved covariates and other artifacts in our datasets. However, statistical testing (t-test with Bonferroni correction) showed that none of these surrogate variables were significantly different between *CHD8^+/−^* cases and matched controls, or between 16p11.2del case and matched controls (p > 0.05), and therefore do not confound our reported signatures. This again suggests that although hidden covariates and other artifacts are present in the data beyond the confounders we already accounted for (sex, age, batch, cell type composition), the observed inflationary pattern in the two signature CpG sets may be attributed to other factors.

We speculate that one such contributing factor may be the presence of groups of highly correlated CpGs (such as those appearing in the same gene promoters), which is quite typical in epigenome-wide studies. Whereas in GWAS the goal is often to identify individual disease-associated SNPs, in EWAS the epigenetic changes typically affect whole genomic regions, hence it is to be expected that multiple CpGs should be detected in the affected region. Such groups of true-positive differentially-methylated CpGs contribute to the tails of the statistical distribution, creating an appearance of an inflationary pattern. In fact van Iterson *et al.* (2017) discussed this effect, and further pointed out that the values of the genomic inflation factor (λ) are typically much higher in EWAS, such that λ in the range 1.33 - 1.72 appeared even for well-calibrated EWAS studies.

With these considerations in mind, our approach was to apply a Mann-Whitney U test to each CpG independently from the *limma* regression models in order to overcome possible inflation of the distribution tails. This non-parametric test is based on comparing relative ranks rather than the magnitude of deviation from the mean, and is therefore relatively robust to unwanted inflation in the tails of the (parametric) distribution. As such, Mann-Whitney test was applied as an additional statistical filter to reduce the number of potential false-positive CpGs in our signature sets: only the CpGs that were identified independently by *limma* regression model and passed the non-parametric test were added to the signature sets.

Overall, we believe that the epigenetic signature sets identified in this study represent a novel approach to detect useful epigenetic patterns with a diagnostic potential in *CHD8^+/−^* and 16p11.2del cohorts. However, there are limitations to this study related to its modest size and other factors, which we acknowledge in our Discussion section. The robustness of our results may be improved further if additional data become available.

References

van Iterson M, van Zwet EW, Consortium B, Heijmans BT. Controlling bias and inflation in epigenome- and transcriptome-wide association studies using the empirical null distribution. Genome biology. 2017;18(1):19.

Leek JT, Johnson WE, Parker HS, Jaffe AE, Storey JD. The sva package for removing batch effects and other unwanted variation in high-throughput experiments. Bioinformatics. 2012;28(6):882–3.
