# Supplementary material for: Functional DNA methylation signatures for autism spectrum disorder genomic risk loci: 16p11.2 deletions and CHD8 variants
Source: Clin Epigenetics. 2019 Jul 16;11:103. doi: 10.1186/s13148-019-0684-3 (PMC6636171; doi:10.1186/s13148-019-0684-3)
Supplement: Supplementary file 2 — Figure S1. Data analysis flowchart. Outline of the training cohorts and analysis pipeline used to derive DNAm signatures and test cohorts (independent samples), on which the DNAm signatures were tested. Figure S2. Identification of differentially methylated CpG sites when comparing 16p11.2del signature cases with age-, sex-matched controls. Significant sites for the DNAm signature for 16p11.2del represented by the overlap from limma regression and Mann Whitney U analysis are shown. Each volcano plot represents the distribution of significant sites at various statistical parameters (y-axis is negative log q-value, Benjamini-Hochberg corrected) and Δβ differences (x-axis is the average difference in DNAm between 16p11.2del and controls). Red horizontal line represents q < 0.05, red vertical lines represent |Δβ| ≥ 5%. Figure S3. Identification of differentially methylated CpG sites when comparing CHD8+/− signature cases with age-, sex-matched controls. Significant sites for the DNAm signature for CHD8+/− represented by the overlap from limma regression and Mann Whitney U analysis are shown. Each volcano plot represents the distribution of significant sites at various statistical parameters (y-axis is negative log q-value, Benjamini-Hochberg corrected) and Δβ differences (x-axis is the average difference in DNAm between CHD8+/− and controls). Red horizontal line represents q < 0.05, red vertical lines represent |Δβ| ≥ 5%. Figure S4. Targeted sodium bisulfite pyrosequencing of selected 16p11.2del and CHD8+/− DNAm signature CpG sites overlapping differentially methylated regions (DMRs). Specific CpG sites from our DNAm signatures that overlapped DMRs found using bump hunting were selected for targeted sodium bisulfite pyrosequencing validation in signature cases and age-, sex-matched controls. In the 16p11.2del group, the following CpG sites were validated: A) cg00108944 and cg23588049 in GLIPR1L2 showing a gain of methylation, B) cg25983544 and cg06377543 in PSMA8 showin [file 13148_2019_684_MOESM2_ESM.pptx]

## Slide 1
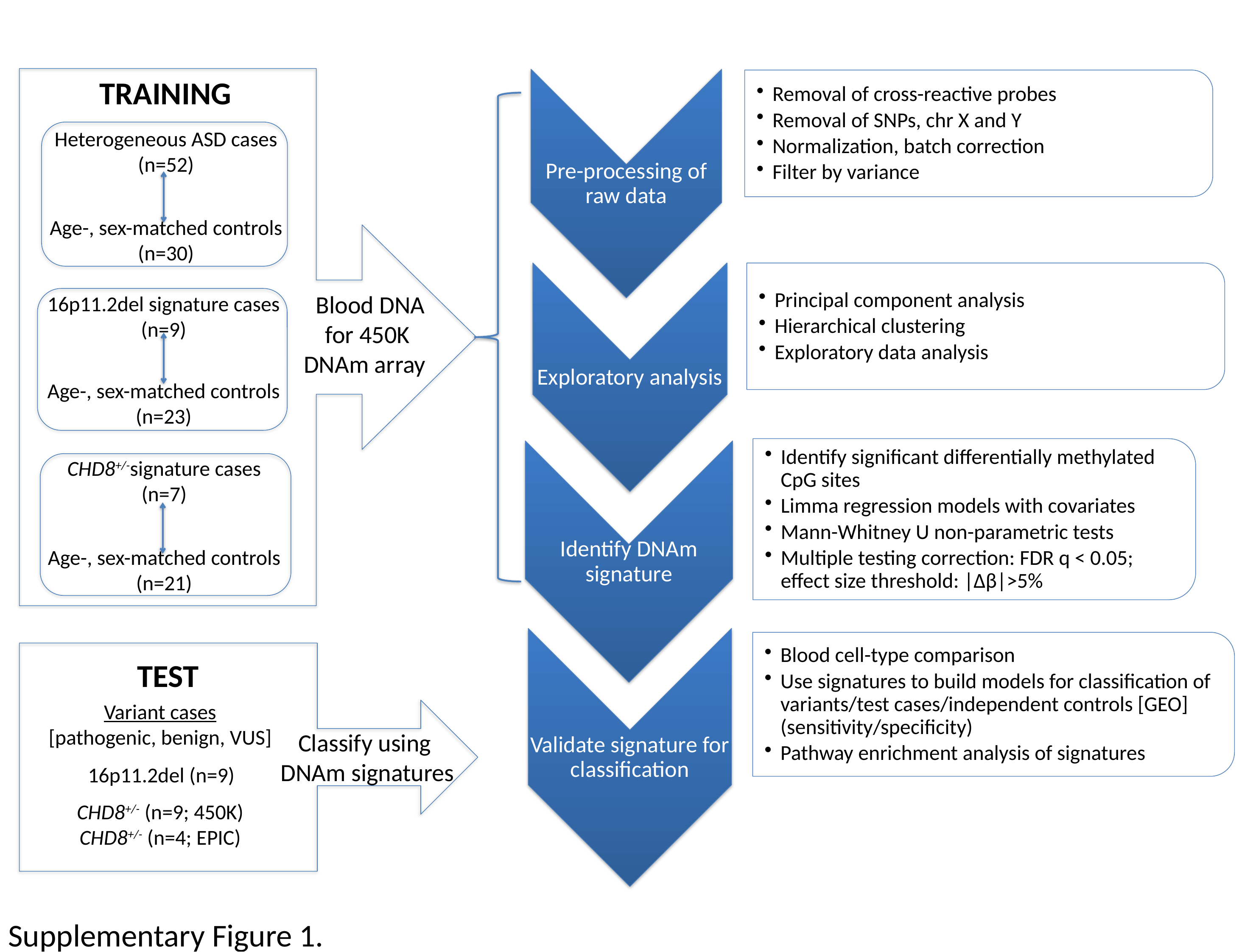

TRAINING
Heterogeneous ASD cases
(n=52)
Age-, sex-matched controls
(n=30)
Blood DNA
for 450K
DNAm array
16p11.2del signature cases
(n=9)
Age-, sex-matched controls
(n=23)
CHD8+/-signature cases
(n=7)
Age-, sex-matched controls
(n=21)
TEST
Variant cases
[pathogenic, benign, VUS]
Classify using
DNAm signatures
16p11.2del (n=9)
CHD8+/- (n=9; 450K)
CHD8+/- (n=4; EPIC)
Supplementary Figure 1.

## Slide 2
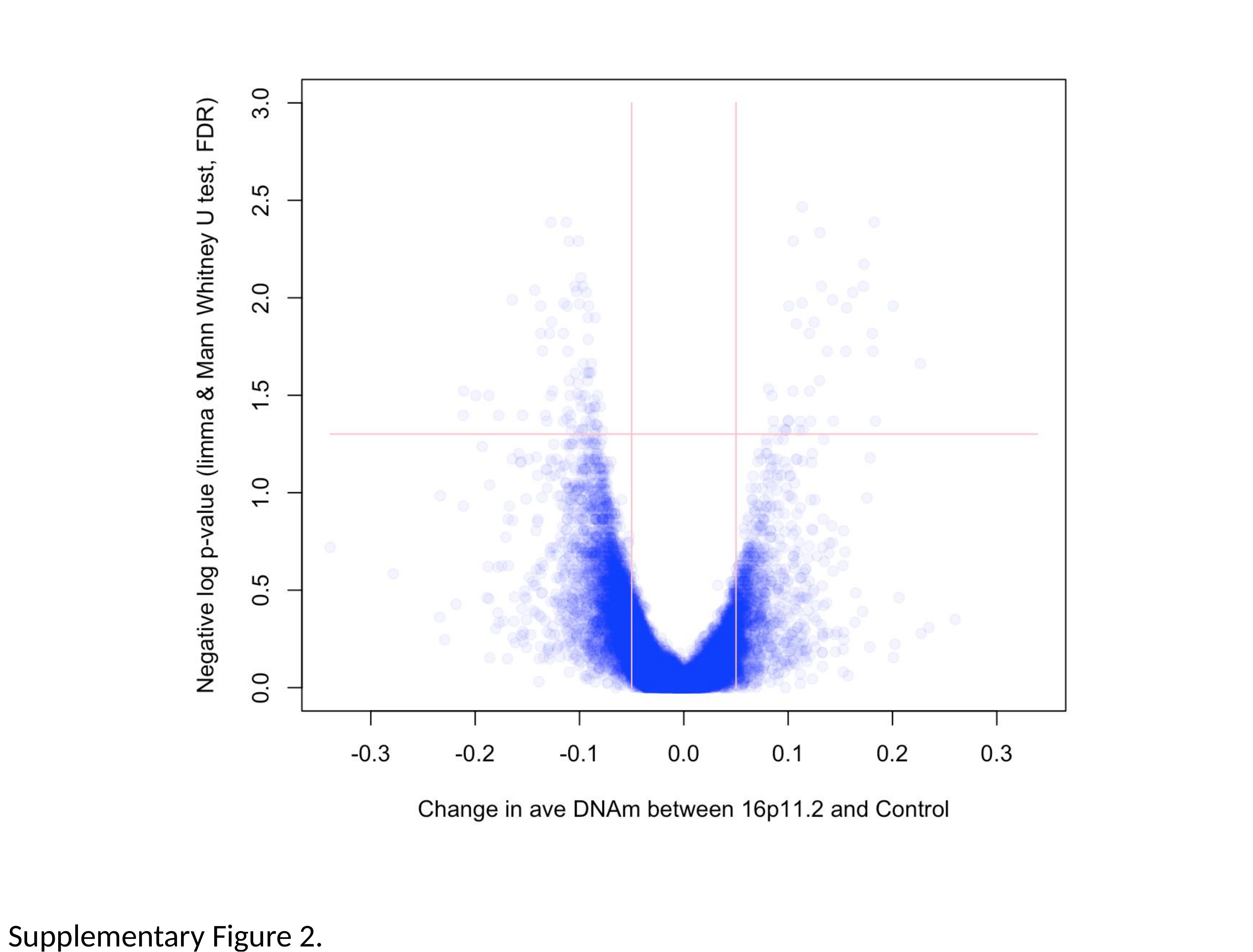

Supplementary Figure 2.

## Slide 3
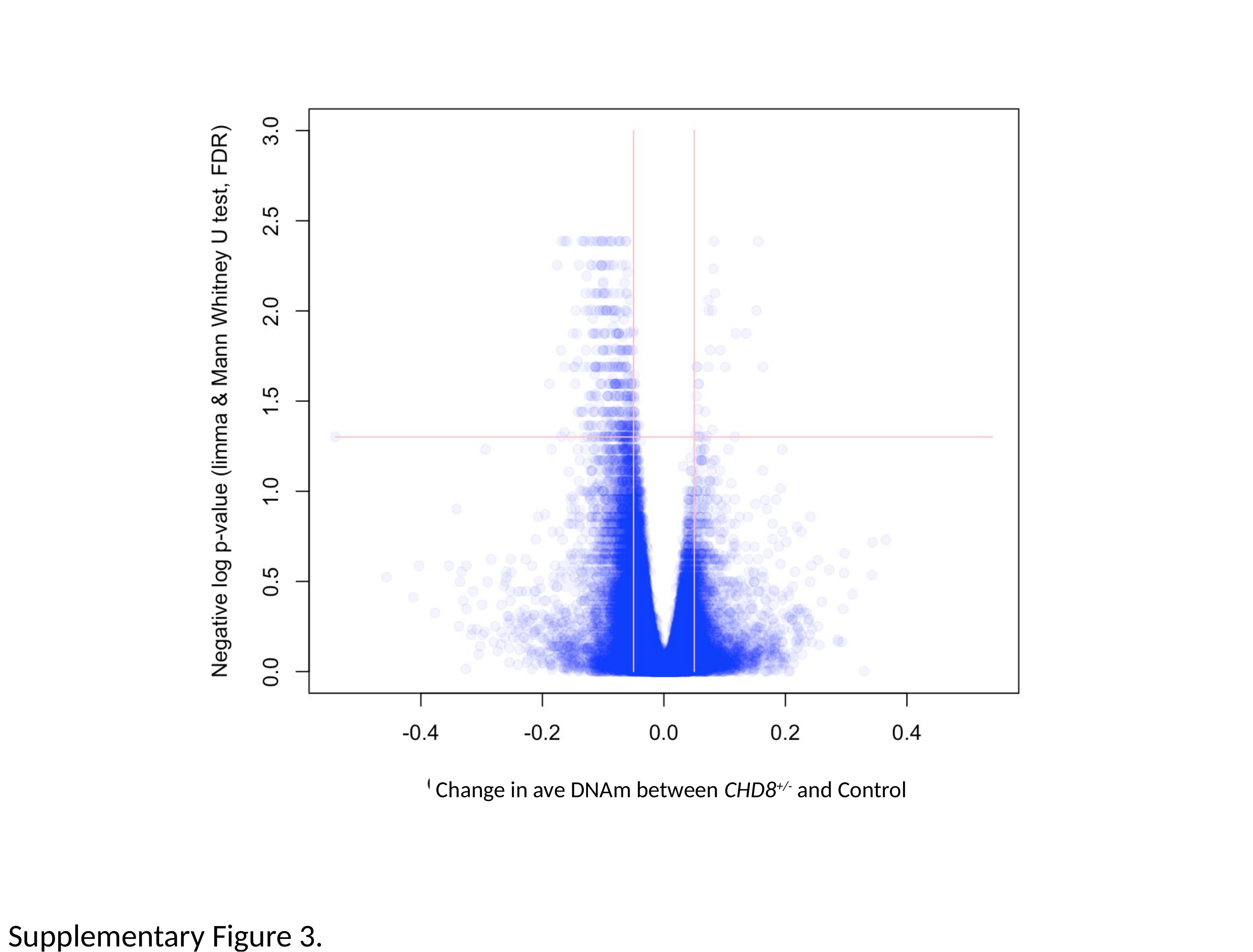

Change in ave DNAm between CHD8+/- and Control
Supplementary Figure 3.

## Slide 4
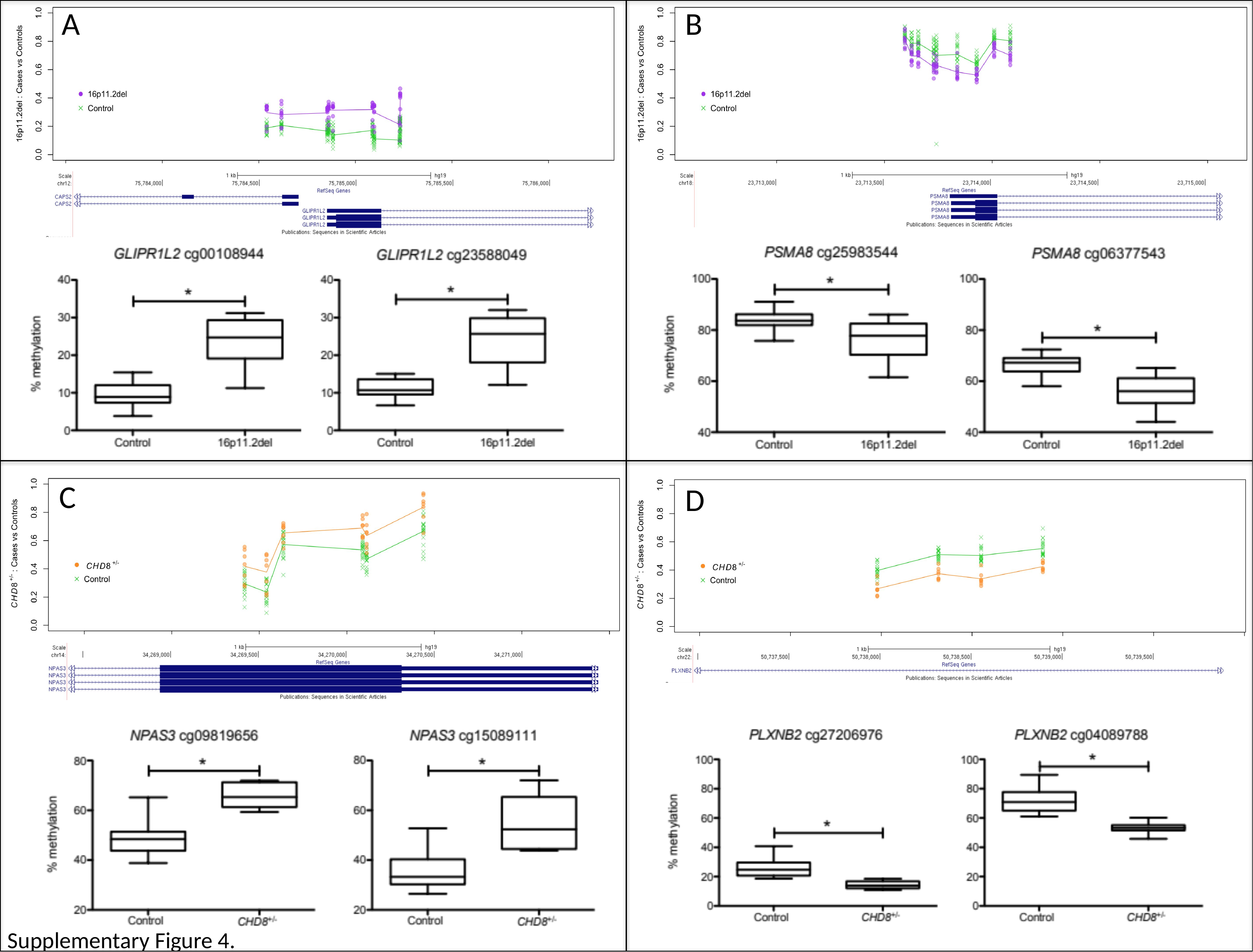

A
B
C
D
Supplementary Figure 4.

## Slide 5
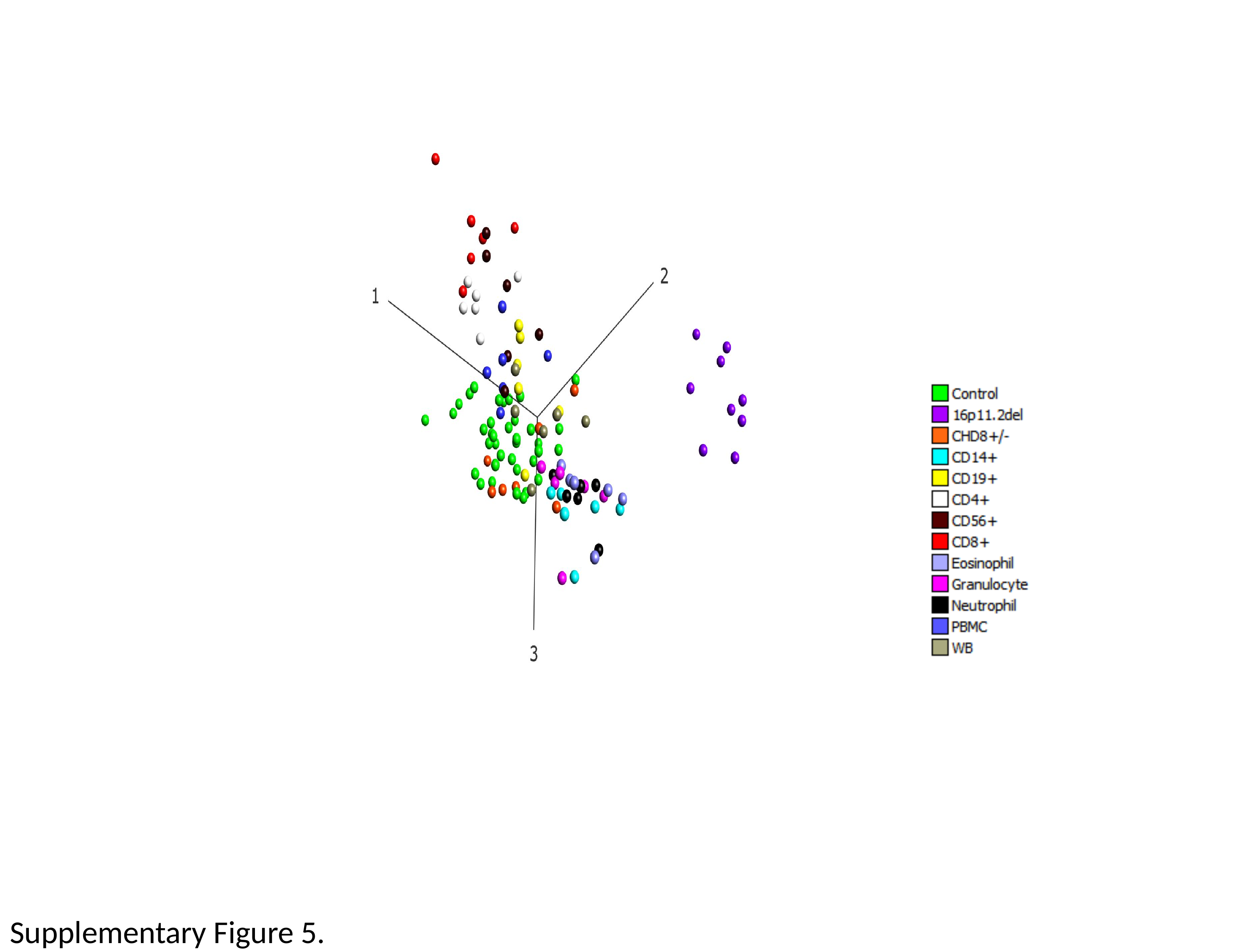

Supplementary Figure 5.

## Slide 6
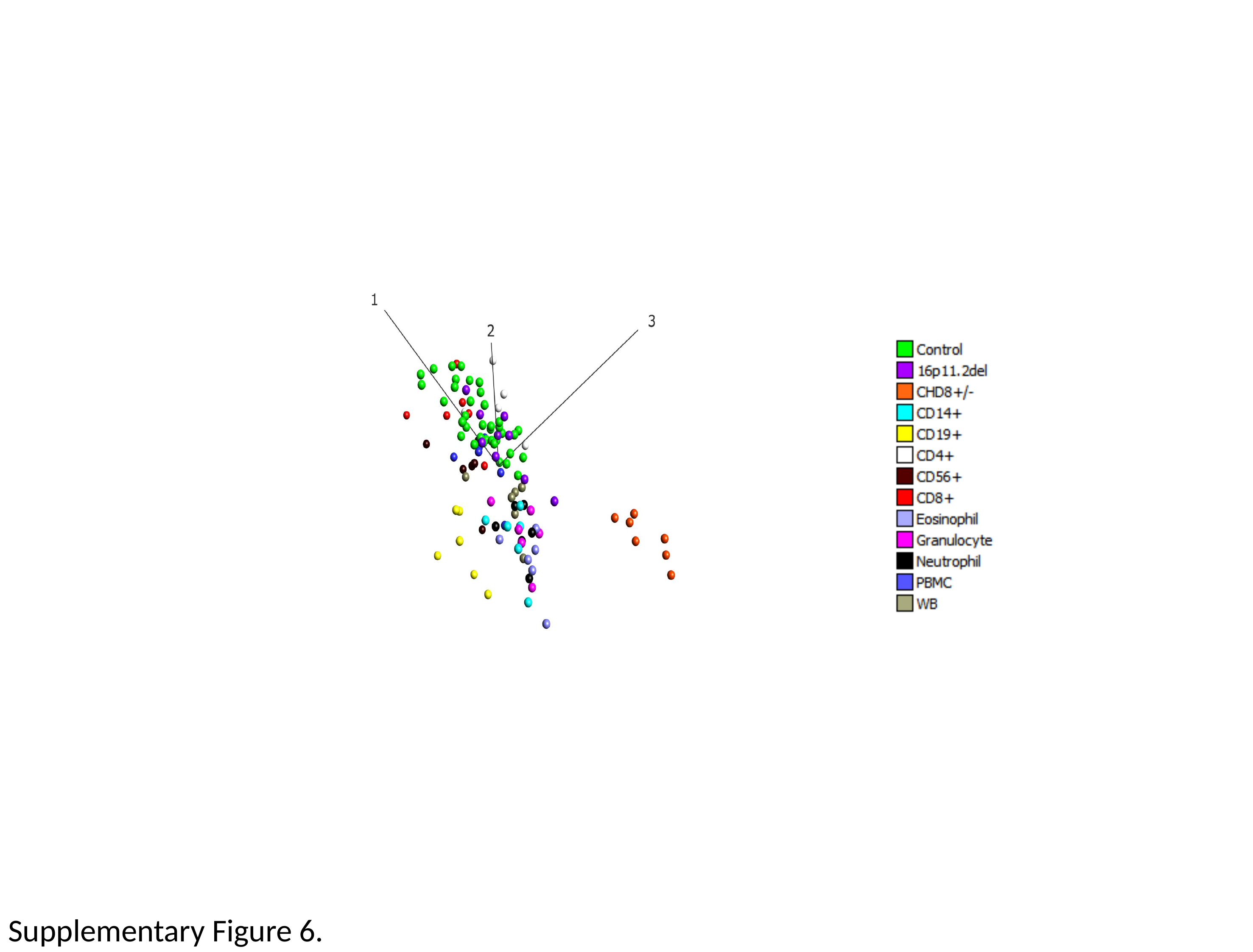

Supplementary Figure 6.

## Slide 7
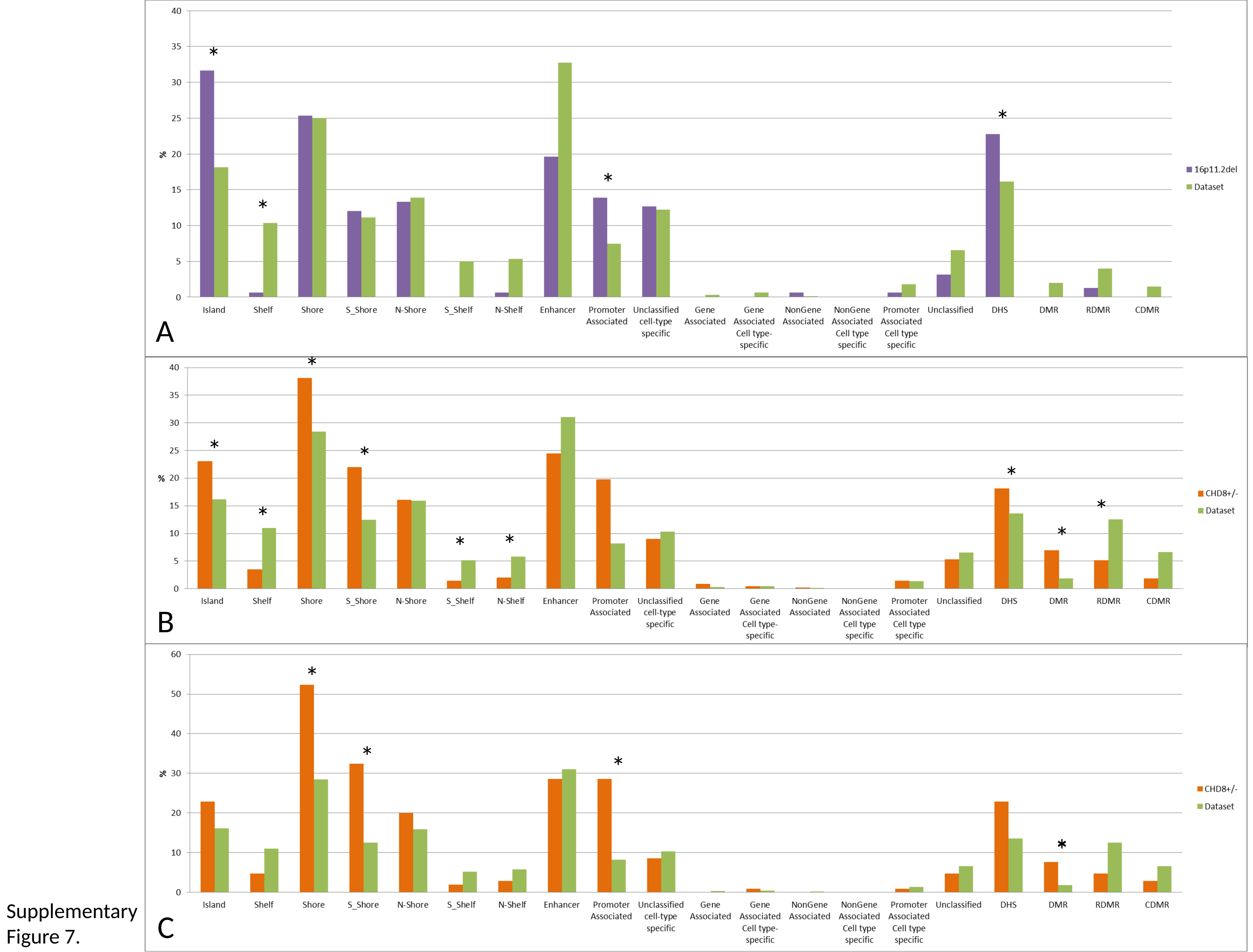

*
*
*
*
A
*
*
*
*
*
*
*
*
*
B
*
*
*
*
*
Supplementary Figure 7.
C

## Slide 8
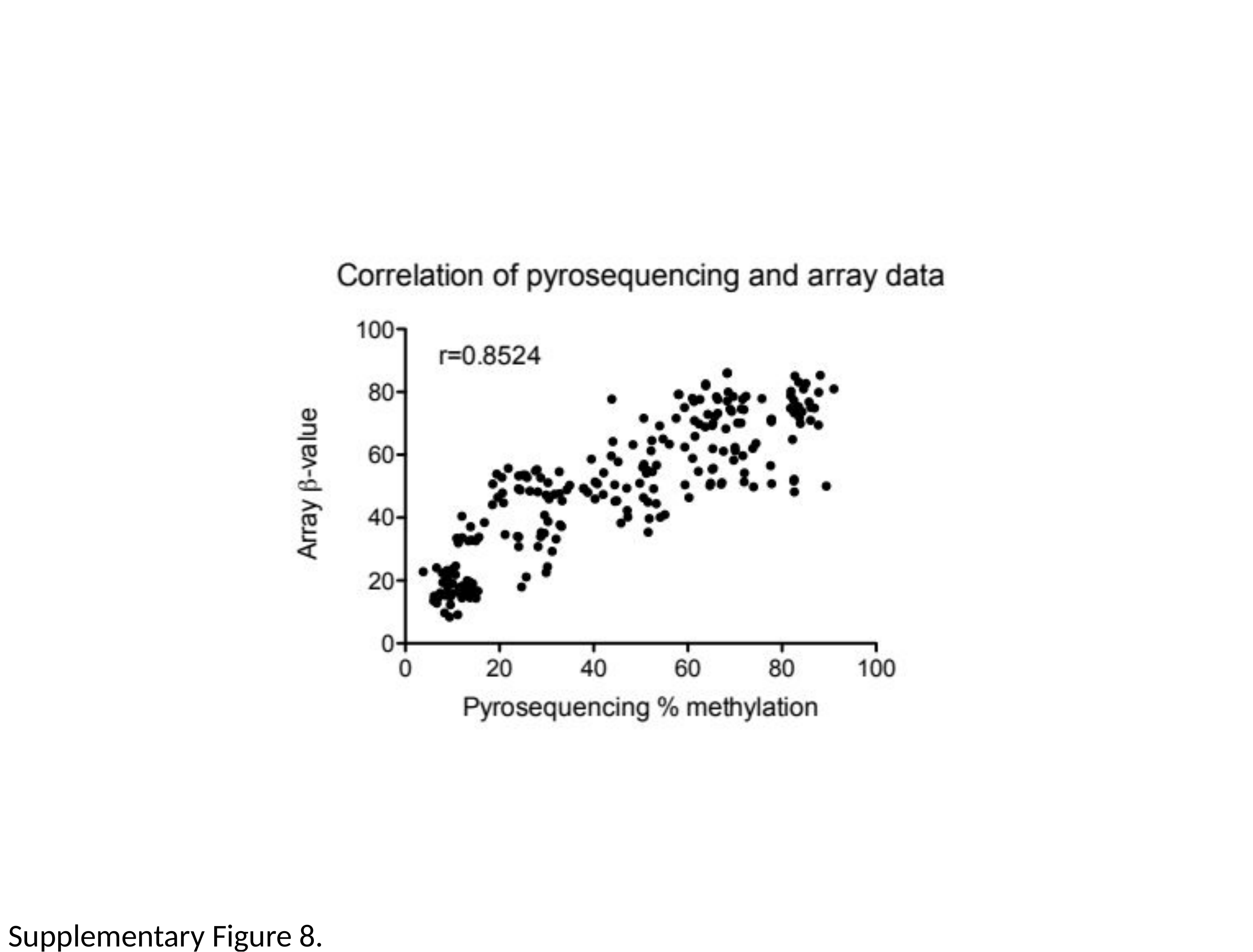

Supplementary Figure 8.

## Slide 9
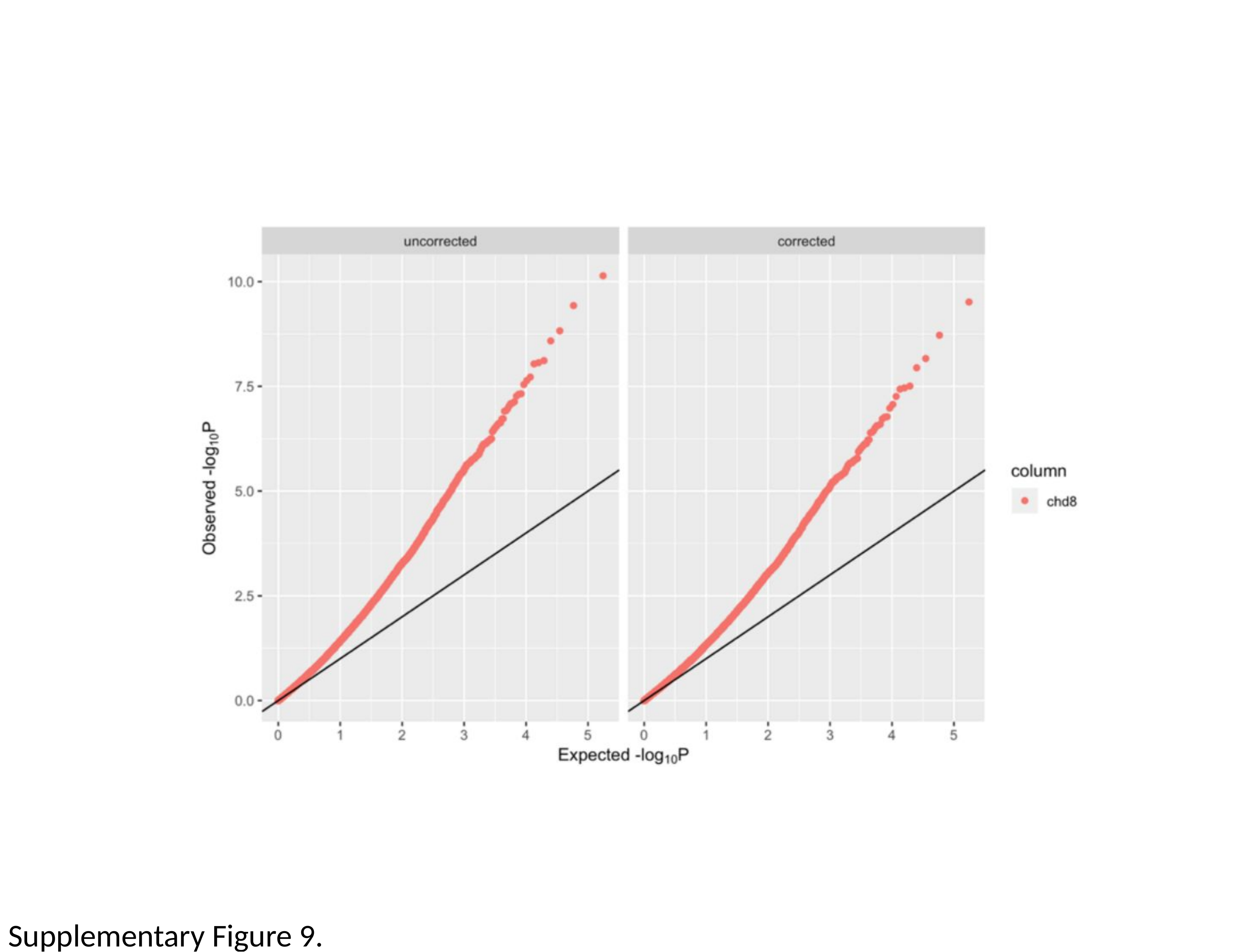

Supplementary Figure 9.

## Slide 10
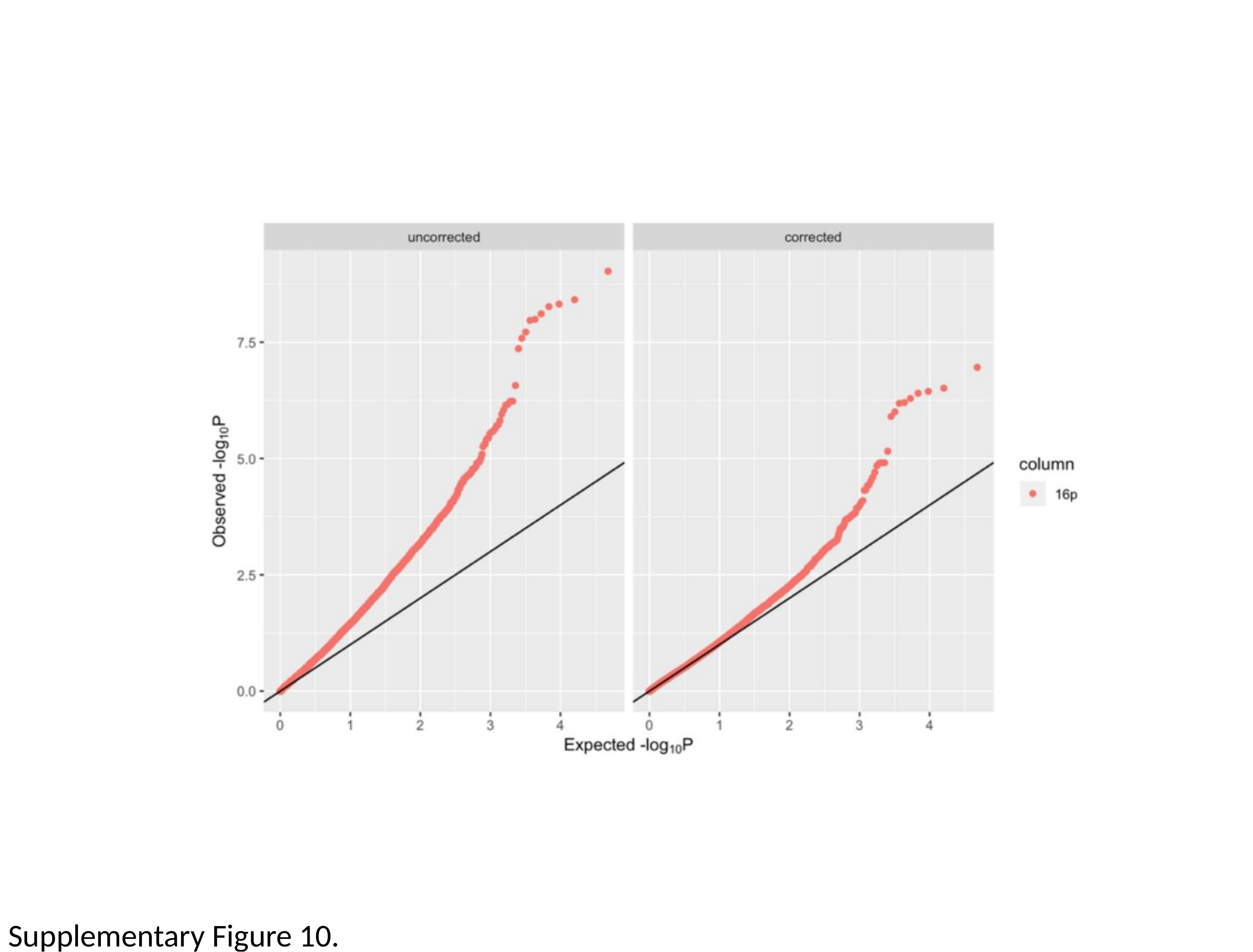

Supplementary Figure 10.
